# Circadian regulation in human white adipose tissue revealed by transcriptome and metabolic network analysis

**DOI:** 10.1038/s41598-019-39668-3

**Published:** 2019-02-25

**Authors:** Skevoulla Christou, Sophie M. T. Wehrens, Cheryl Isherwood, Carla S. Möller-Levet, Huihai Wu, Victoria L. Revell, Giselda Bucca, Debra J. Skene, Emma E. Laing, Simon N. Archer, Jonathan D. Johnston

**Affiliations:** 1 Faculty of Health and Medical Sciences, University of Surrey, Guildford, UK; 2 Present Address: Department of Medicine, Brigham and Women’s Hospital, Boston, USA; 3 Bioinformatics Facility, Faculty of Health and Medical Sciences, University of Surrey, Guildford, UK; 4 Present Address: School of Pharmacy and Biomolecular Sciences, University of Brighton, Brighton, UK

## Abstract

Studying circadian rhythms in most human tissues is hampered by difficulty in collecting serial samples. Here we reveal circadian rhythms in the transcriptome and metabolic pathways of human white adipose tissue. Subcutaneous adipose tissue was taken from seven healthy males under highly controlled ‘constant routine’ conditions. Five biopsies per participant were taken at six-hourly intervals for microarray analysis and *in silico* integrative metabolic modelling. We identified 837 transcripts exhibiting circadian expression profiles (2% of 41619 transcript targeting probes on the array), with clear separation of transcripts peaking in the morning (258 probes) and evening (579 probes). There was only partial overlap of our rhythmic transcripts with published animal adipose and human blood transcriptome data. Morning-peaking transcripts associated with regulation of gene expression, nitrogen compound metabolism, and nucleic acid biology; evening-peaking transcripts associated with organic acid metabolism, cofactor metabolism and redox activity. *In silico* pathway analysis further indicated circadian regulation of lipid and nucleic acid metabolism; it also predicted circadian variation in key metabolic pathways such as the citric acid cycle and branched chain amino acid degradation. In summary, *in vivo* circadian rhythms exist in multiple adipose metabolic pathways, including those involved in lipid metabolism, and core aspects of cellular biochemistry.

## Introduction

Many aspects of mammalian metabolism exhibit daily variation driven in part by an endogenous circadian timing system^1^. This system is comprised of a central ‘master’ clock in the hypothalamic suprachiasmatic nuclei and an integrated network of circadian clocks present in all major tissues within the body^2^. Circadian disruption causes abnormal metabolic physiology^3^. Misalignment of human clocks with each other and the environment is believed to be a major contributor to obesity and related pathologies associated with shift work^4,5^.

At the molecular level, circadian clocks are formed from a set of inter-locking transcriptional translational feedback loops (TTFLs). In the core TTFL, transcription factors CLOCK and ARNTL (also known as BMAL1) stimulate transcription of three *Period* (*Per*) and two *Cryptochrome* (*Cry*) genes. Once translated the PER and CRY proteins bind to one another, translocate to the nucleus and inhibit the transcriptional activity of CLOCK and BMAL1, eventually repressing their own transcription^6^. A key secondary loop involves rhythmic transcription of *Rev-erbα* (*Nr1d1*) by the CLOCK-BMAL1 complex. The resulting rhythmic accumulation of REVERBα protein provides temporal inhibition of *Bmal1* transcription and thus feeds back onto the core TTFL^7^. In addition to defining internal biological time, clock proteins also bind to response elements in output genes^8,9^. Many of these output genes themselves encode transcription factors and thus the circadian clock can temporally regulate a large part of the transcriptome^10,11^.

It is estimated that nearly half of all murine genes exhibit a circadian expression profile in at least one tissue of the body^10^. Many of the rhythmic genes in a tissue are integral to local physiological function. Despite advances in understanding of circadian rhythmicity in animal tissues, there is very little information on molecular rhythms in humans. Transcriptomic analyses of human whole blood suggest that approximately 7–9% of genes in whole blood RNA are rhythmic^12,13^, a similar proportion to that found within individual mouse tissues. However, it is difficult to access other human tissues for serial sampling. We and others have recently developed biopsy protocols for serial tissue collection of human skeletal muscle and subcutaneous white adipose tissue over 24-hours^14–16^. Here we have combined our adipose tissue biopsy method with the gold standard ‘constant routine’ protocol^17^ to study the circadian transcriptome in human adipose tissue. The constant routine protocol removes rhythmic changes in environmental factors and behaviours (including light-dark, sleep-wake and feed-fast cycles) that are known to influence human circadian rhythms^13,18^. Resulting rhythms are therefore driven primarily by the endogenous circadian system.

## Results

In this study seven healthy male participants underwent restricted sleep-wake and meal times before entering the laboratory, to maximise circadian synchronisation. They maintained this sleep and feeding schedule for three days in the laboratory before undergoing a 37-hour constant routine, during which five six-hourly subcutaneous adipose tissue biopsies were taken per participant. Transcriptomic data were generated from these biopsies and then subjected to in-depth bioinformatic analysis.

### Circadian expression of the human adipose transcriptome

**Figure 1:**
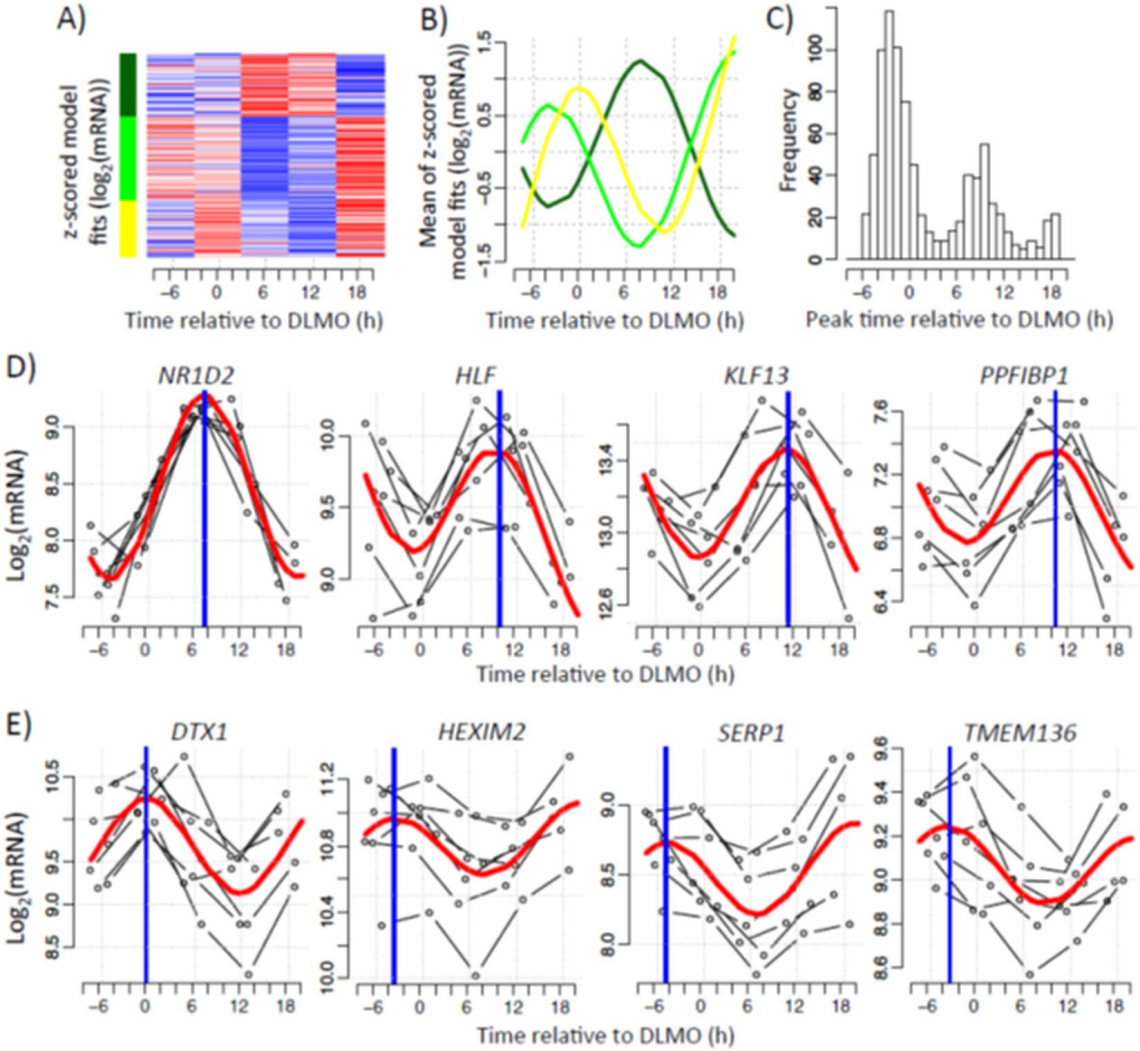
Circadian expression of the human white adipose tissue transcriptome. Temporal expression profiles measured in five six-hourly white adipose tissue biopsies collected from seven participants in constant routine conditions. (**A**) Heatmap showing the mixed model values at averaged DLMO times (average per sampling point across subjects). Red represents high expression, blue low expression. (**B**) The average of z-scored mixed model profiles per cluster was calculated. Lines represent the average profile of all probes within each cluster. (**C**) Histogram of peak time estimates of probes identified as exhibiting circadian rhythmicity. Peak times were derived from the mixed modelling. (**D**,**E**) Representative profiles of probes exhibiting either (**D**) morning and (**E**) evening peak expression. Thin black lines show each participant’s gene expression profile plotted according to their own DLMO. The red thick line depicts the sinusoidal model fit and the blue thick vertical line indicates estimated peak time.

Genes were classified as circadian if the expression profile(s) of one or more of their associated transcripts exhibited one full oscillation every 24 hours. To identify the set of genes with circadian profiles we fitted a sinusoidal function to the data, set a threshold to the R^2^ value of the fit and restricted the amplitude’s 95% CI to not include zero. Using these criteria, we identified circadian rhythms in 837 transcripts (~2% of all transcript targeting probes). Unsupervised clustering performed on the 837 circadian transcripts revealed 3 distinct clusters (Fig. 1A). Two of these clusters (yellow, 233 transcripts; light green, 346 transcripts) had peak expression in the circadian evening, whereas the third cluster (dark green, 258 transcripts) had peak expression in the circadian morning (Fig. 1B). Furthermore, a frequency plot of the peak time of each rhythmic transcript revealed a bimodal distribution, with peaks predominantly occurring in the evening and morning (Fig. 1C). For subsequent analysis, yellow and light green clusters were pooled together to form a set of 579 ‘evening’ transcripts, whereas the dark green cluster provided the 258 ‘morning’ transcripts. Example transcript profiles for morning and evening-peaking genes are presented in Fig. 1D,E, respectively. A full list of all circadian transcripts is provided in Supplementary Table S1. The 837 transcripts are associated with 727 unique genes, on which we focus our interpretation.

### Function of circadian genes in the human adipose transcriptome

**Figure 2:**
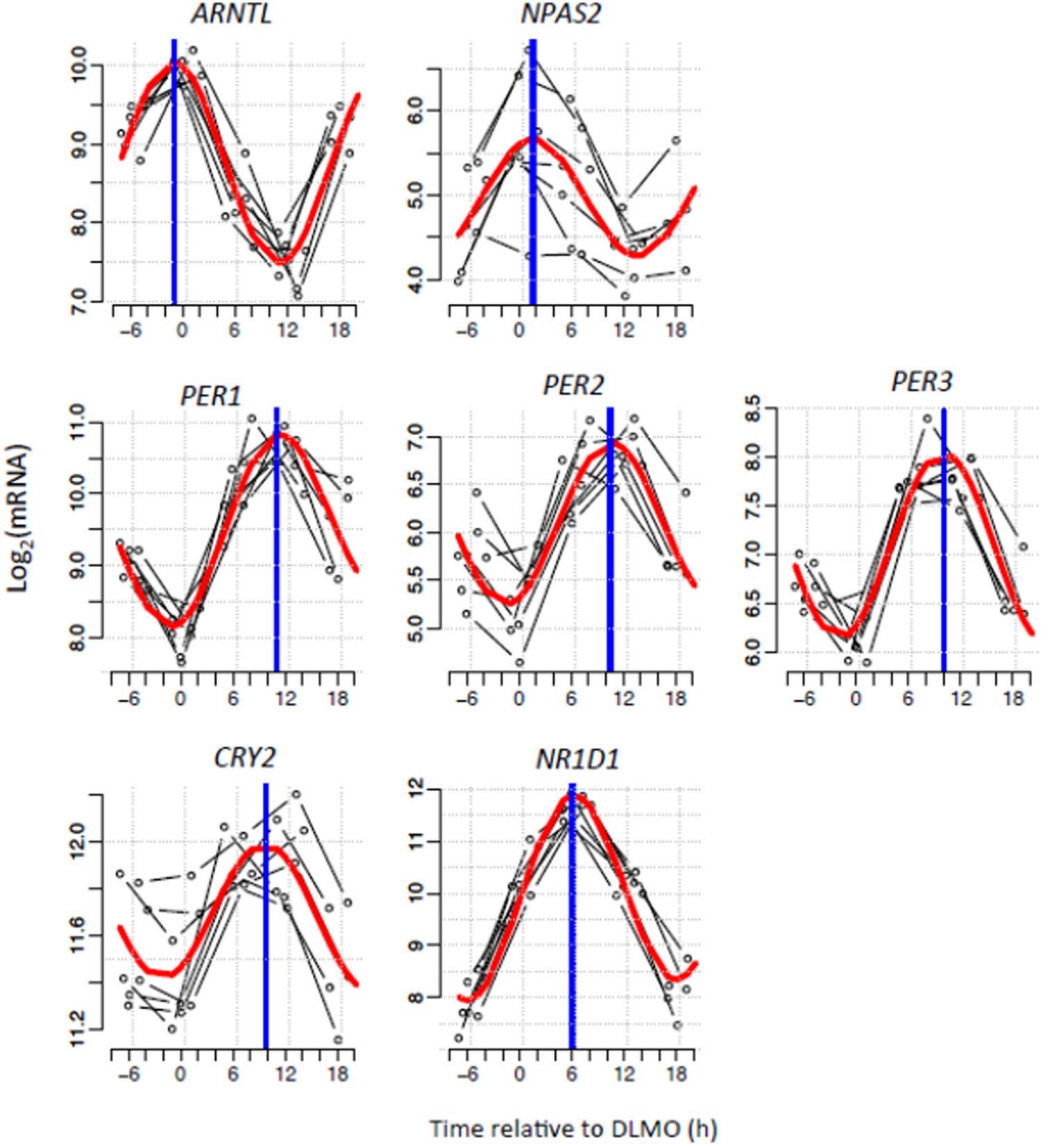
Circadian rhythms of canonical clock genes in human white adipose tissue. Temporal expression profiles of canonical clock genes measured in five six-hourly white adipose tissue biopsies collected from seven participants in constant routine conditions. Thin black lines show each participant’s gene expression profile plotted relative to their own DLMO. The red thick line depicts the sinusoidal model fit and the blue thick vertical line indicates the estimated peak time.

Canonical clock genes exhibited robust rhythmicity in biopsies taken under constant routine conditions. Seven core clock genes were found to be rhythmic. Two clock genes (*ARNTL*, *NPAS2*) peaked in the circadian evening; the other clock genes (*PER1*, *PER2*, *PER3*, *CRY2*, *NR1D1*) peaked in the circadian morning (Fig. 2).

Validated databases were interrogated using our 727 circadian genes to identify rhythmic biological functions. GO enrichment analysis revealed a temporal separation of biological processes associated with circadian genes (Table 1). Most of the processes associated with the evening peaking transcripts were aspects of metabolism, including organic acid, co-factor and oxidation-reduction. Morning peaking genes were significantly associated with gene expression regulation, nucleic acid regulation and other metabolic processes. Further GO molecular function enrichment analysis indicated a predominance of nucleic acid and transcription factor binding in the morning, whereas catalytic and reductase activity was enhanced in the evening (Table 2).

**Table 1:** GO (process) enrichment of human adipose circadian genes.

Morning (Cluster 1) 258 probes			Evening (Cluster 2 and 3) 579 probes		
Biological Process	p-value	FDR	Biological Process	p-value	FDR
Gene expression	3.609E-08	1.023E-04	Single-organism metabolic process	1.846E-18	8.853E-15
Cellular nitrogen compound metabolic process	2.876E-07	4.077E-04	Organonitrogen compound metabolic process	7.917E-15	1.300E-11
Histone H3 deacetylation	4.844E-07	4.577E-04	Cofactor metabolic process	8.134E-15	1.300E-11
RNA metabolic process	1.756E-06	1.245E-03	Small molecule metabolic process	2.904E-14	3.481E-11
Nitrogen compound metabolic process	3.328E-06	1.887E-03	Organic acid metabolic process	9.592E-14	9.199E-11
Nucleobase-containing compound metabolic process	4.157E-06	1.964E-03	Carboxylic acid metabolic process	5.182E-13	4.141E-10
Circadian regulation of gene expression	5.557E-06	2.251E-03	Oxoacid metabolic process	6.827E-13	4.676E-10
Cellular metabolic process	7.245E-06	2.568E-03	Coenzyme metabolic process	2.214E-12	1.327E-09
Heterocycle metabolic process	1.083E-05	3.411E-03	Single-organism biosynthetic process	8.464E-12	4.509E-09
Cellular aromatic compound metabolic process	1.703E-05	4.613E-03	Oxidation-reduction process	1.589E-10	7.618E-08

**Table 2:** GO (molecular function) enrichment of human adipose circadian transcripts.

Morning cluster (Cluster 1) 258 probes			Evening cluster (Cluster 2 and 3) 579 probes		
Molecular Function	p-value	FDR	Molecular Function	p-value	FDR
Heterocyclic compound binding	3.255E-07	1.327E-04	Catalytic activity	2.554E-12	2.521E-09
Organic cyclic compound binding	5.835E-07	1.327E-04	Oxidoreductase activity	1.826E-09	9.012E-07
Nucleic acid binding	2.277E-06	3.454E-04	Binding	5.370E-08	1.767E-05
Transcription corepressor binding	4.662E-05	4.315E-03	Protein binding	5.370E-06	1.325E-03
Transcription cofactor binding	4.741E-05	4.315E-03	Transferase activity, transferring alkyl or aryl (other than methyl) groups	9.583E-06	1.892E-03
Core promoter binding	9.358E-05	7.097E-03	Oxidoreductase activity, acting on the CH-NH group of donors, NAD or NADP as acceptor	1.455E-05	2.394E-03
Binding	3.083E-04	2.004E-02	Lyase activity	2.189E-05	2.802E-03
RNA binding	5.542E-04	3.011E-02	Electron carrier activity	2.271E-05	2.802E-03
Ubiquitin binding	6.378E-04	3.011E-02	Aldehyde-lyase activity	7.113E-05	7.800E-03
Core promoter sequence-specific DNA binding	6.617E-04	3.011E-02	Carbon-carbon lyase activity	1.021E-04	1.008E-02

### Comparison with the adipose circadian/diurnal transcriptome in non-human species

In a previous study of mouse white adipose tissue, 856 genes (~4% of genes assessed) were identified as having circadian rhythmicity^10^. Of those, only 32 transcripts were also classified as rhythmic in our human data set (Supplementary Table S2): 19 peaked in the evening and included the positive arm canonical clock genes, *Bmal1* and *Npas2*, whilst the remaining 13 peaked in the morning. Phases within the mouse data were expressed relative to circadian time (CT). The acrophases of most of these genes occurred at similar times within the behavioural cycle of diurnal humans and nocturnal mice. For example, *PER3* in our human data peaks at 8.8 hours after the dim light melatonin onset (DLMO), which is near the time of awakening, whilst in the mouse white adipose tissue data *Per3* peaked at CT12, which is defined as the onset of activity in constant darkness (i.e. biological evening for a nocturnal rodent). Similarly, the antiphasic gene *ARNTL* (*BMAL1*) in humans had an acrophase of −0.9 hours relative to the DLMO, which equates to biological evening, whilst in mouse white adipose tissue it peaked at CT23 which approximates to the onset of the main rest phase (biological morning in a nocturnal rodent). A summary of the phases is presented in Supplementary Table S2.

We also compared the circadian rhythmic genes from our data set with genes expressed in baboon white adipose tissue that have recently been reported to have diurnal rhythmicity, i.e. 24-hour rhythms when experimental subjects are in an entrained rhythmic environment^19^. Only 14 genes were rhythmic in both human and baboon adipose data sets: *CRY2*, *PER1*, *PER2*, *NPAS2*, *ARNTL*, *NR1D1*, *ZDHHC14*, *SCN3B*, *PCYT2*, *TMEM8A*, *GNA12*, *P4HA2*, *C3orf31* (*TAMM41*) and *RASL10B*.

**Figure 3:**
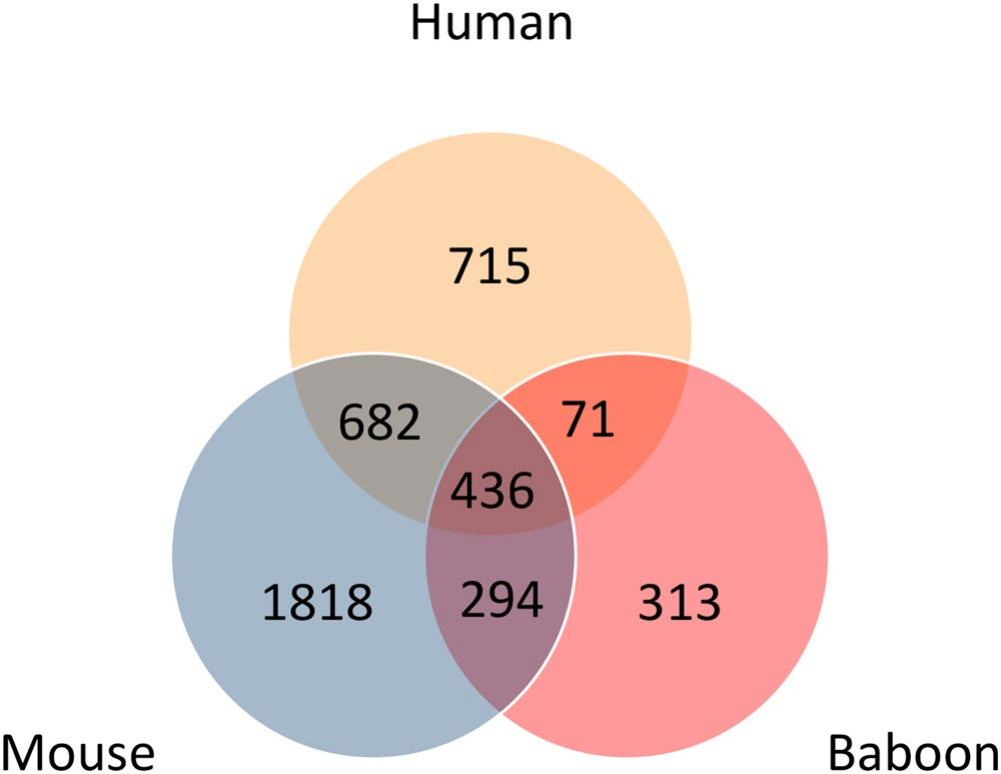
Overlap of Gene Ontology biological process terms associated with rhythmically expressed genes in human, mouse and baboon. Only terms associated with rhythmic genes for which there is a homolog in human considered.

Because of the limited overlap in rhythmic genes between the human and mouse and baboon tissues, we also investigated the overlap in GO biological processes associated with the human adipose rhythmic genes, which could potentially show higher levels of similarity. GO annotation for all three genomes (human, mouse, and baboon) was downloaded from the Gene Ontology database (https://www.ebi.ac.uk/GOA/ on 13 October 2018). From these annotations the GO terms associated with biological processes were retained. Rhythmic genes that are homologous to (or present in) human were annotated using the respective GO biological process annotation. Overlaps in the identifiers of the GO terms was then assessed. The results of this analysis are shown in the Venn diagram of Fig. 3. The mouse has the largest number of associated GO terms and the baboon the least, and these differences presumably reflect differences in the depths of annotation for each species. However, greater levels of overlap are now apparent with 27% of human biological processes overlapping with 46% of the baboon processes, and 59% of human biological processes overlapping with 35% of the mouse processes. Four hundred and thirty-six terms were common to each species, which represents 23%, 39% and 14% of the human, baboon and mouse total biological processes, respectively. Supplementary Table S3 shows the top ten biological processes for the human vs. baboon, human vs. mouse and all three species comparisons that have been ranked according to the frequency of occurrence of the human biological processes.

### Comparison with the human whole-blood transcriptome

To assess the similarity of circadian transcriptomes in different human tissues, we compared the current data from human white adipose tissue with published data sets from human whole-blood samples. Specifically, we used data from the constant routine following a week of sufficient sleep in a sleep restriction study^12^ and the baseline condition of a forced desynchrony study where sleep occurred in phase with melatonin^13^. Comparison of the datasets identified a larger overlap between circadian transcriptomes of the adipose tissue and blood samples taken in constant routine, than adipose tissue and blood taken from the baseline period of the forced desynchrony study. In total, 14 genes were circadian in both blood data sets and in human subcutaneous adipose tissue (Supplementary Dataset 1). These genes included the canonical clock genes, *ARNTL (BMAL1)*, *NPAS2*, *PER2*, *PER3*, together with *NR1D2 (REVERB-β)*, ribosome biogenesis factor (*BMS1*), C-type lectin domain family 18 member b (*CLEC18B*), clusterin associated protein 1 (*CLUAP1*), coactosin-like F-actin binding protein 1 (*COTL1)*, histone cluster 2 H2B family member E (*HIST2H2BE*), myelin protein zero-like 1 (*MPZL1)*, poly(A) binding protein interacting protein 2B (*PAIP2B)* and ribosomal protein large subunit 22 (*RPL22*).

### Interaction network analysis

**Figure 4:**
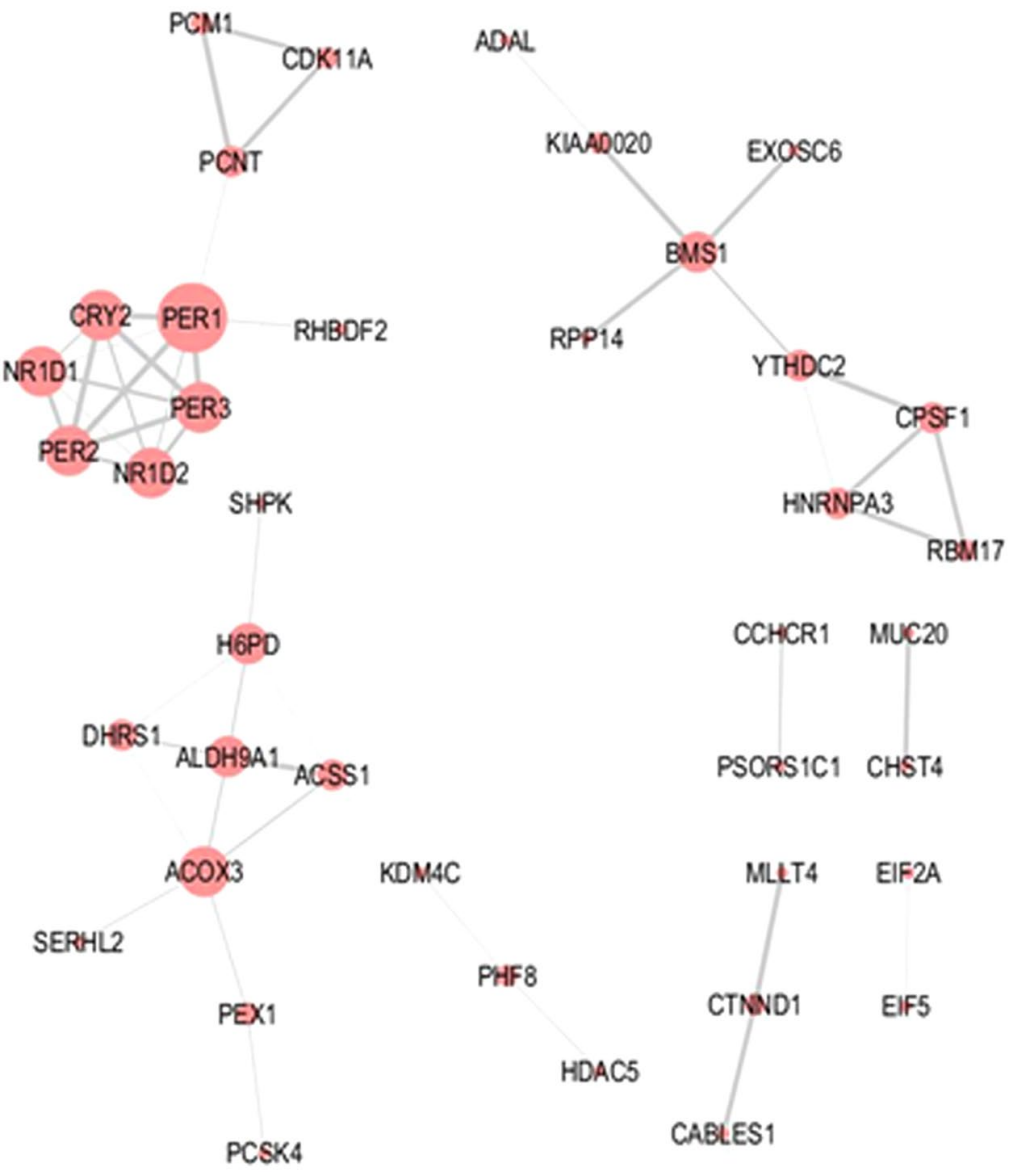
Molecular interaction network associated with morning-peaking genes. Molecular interaction networks for genes encoding transcripts found to peak during the morning were computed using the STRING online database. Node size reflects the number of direct connections a molecule has within the network. Thickness of lines (edges) connecting nodes represents strength of evidence (confidence) supporting each connection as provided by STRING.

In addition to the GO enrichment analyses, predicted molecular interactions related to the 727 circadian genes were assessed using the online STRING tool. Predicted interactions derived from the morning-peaking genes were limited (Fig. 4). The main cluster within the network was associated with the circadian clock. However, small interaction networks were also observed for mRNA processing/splicing (e.g. Cleavage and polyadenylation specificity factor subunit 1, CPSF1; Heterogeneous nuclear ribonucleoprotein A3, HNRNPA3; Splicing factor 45/RNA-binding motif protein 17, RBM17), cell cycle/centrosome regulation (e.g. Cyclin-dependent kinase 11 A, CDK11A; Pericentriolar material 1 protein, PCM1; Pericentrin, PCNT), and oxido-reductase/dehydrogenase activity (e.g. Acyl-CoA oxidase 3, pristanoyl, ACOX3; Acyl-CoA synthetase short chain family member 1, ACSS1; Aldehyde dehydrogenase 9 family member A1, ALDH9A1; Dehydrogenase/reductase 1, DHRS1; Hexose-6-phosphate dehydrogenase, H6PD).

A much larger set of interactions was generated from analysis of the evening-peaking genes (Supplementary Fig. S1). Within this network, many of the molecules with the largest number of interactions were involved in fatty acid, amino acid and carbohydrate metabolism. These included: Short-chain specific acyl-CoA dehydrogenase (SCAD, from the *ACADS* gene), Aldehyde dehydrogenase family 1 member B1 (ALDH1B1), Citrate synthase (CS), Dihydrolipoyllysine-residue acetyltransferase component of pyruvate dehydrogenase complex (DLAT), 3-hydroxyisobutyryl-CoA hydrolase (HIBCH), Malate dehydrogenase (MDH2), and Serine hydroxymethyltransferase (SHMT1).

The network generated from the evening-peaking genes also included some smaller clusters. These included clusters relating to glutathione metabolism (Glutathione S-transferase Mu 1, GSTM1; GSTM3; GTSM4; GTSM5; Glutathione peroxidase 8, GPX8), protein ubiquitination (Ankyrin repeat and SOCS box protein 8, ASB8; F-box only protein 15, FBXO15; Leucine-rich repeat protein 1, LRR1; TNF receptor-associated factor 7, TRAF7; Ubiquitin-conjugating enzyme E2 L3, UBE2L3), protein trafficking and Golgi function (ADP-ribosylation factor 1, ARF1; ARF4; BET1-like protein, BET1L; Coatomer subunit gamma-2, COPG2; Dynein light chain 1, DYNLL1; DYNLL2; KDEL endoplasmic reticulum protein retention receptor 2; KDEL2; Kinesin-1 heavy chain, KIF5B; Surfeit locus protein 4, SURF4).

### Metabolic network analysis

**Figure 5:**
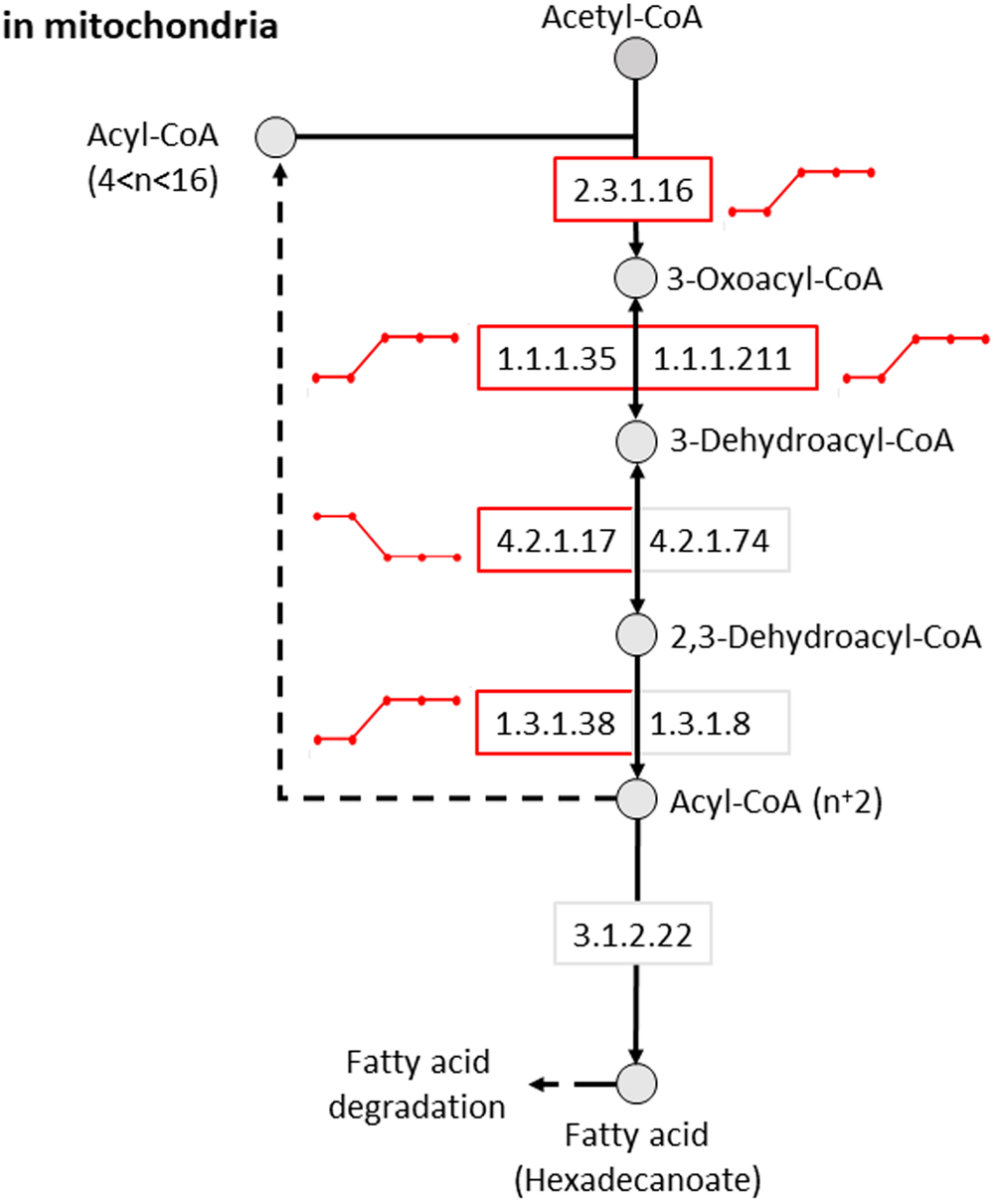
Predicted rhythmicity of the mitochondrial short-chain, fatty acid elongation pathway. The activity of metabolic reactions in a metabolic model was assessed using the ‘Fast iMAT’ algorithm of MUFINS using averaged transcriptome samples and the human metabolic model Recon2 as input. For each reaction within the metabolic model we obtained a predicted metabolic activity profile across the circadian cycle. EC numbers represent specific enzymatic reactions. Circles and boxes indicate metabolites and reactions, respectively. Red boxes indicate reactions that were identified as rhythmic. Lines next to each circadian reaction indicate the dynamics (high or low) for each reaction across each of the five biopsy time points.

To predict circadian variation in metabolic reactions and associated pathways, our transcriptome data were applied to comprehensive pre-existing models of human metabolism. Consistent with our GO analysis, circadian rhythms were closely associated with fatty acid metabolism. Multiple reactions in mitochondrial fatty acid elongation (Fig. 5), biosynthesis and degradation pathways were predicted to exhibit circadian variation.

Analyses also predicted circadian variation in multiple steps of other key cellular metabolic pathways including: purine and pyrimidine metabolism; the citric acid cycle, the pentose phosphate pathway, branched chain amino acid degradation pathways and glycosylphosphatidylinositol (GPI) anchor biosynthesis. The full list of reactions and metabolic pathways that display change in activity can be found in Supplementary Dataset 2 (rhythmic reactions identified by R^2^ ≥ 0.8).

## Discussion

This study contains the first 24-hour transcriptomic profiling of human adipose tissue in samples collected during a protocol that permits expression of endogenous circadian rhythms *in vivo*. Unmasking of circadian rhythms in a constant routine protocol permits removal/minimisation of rhythmic changes in the environment, feeding behaviour and sleep-wake physiology, which are known to influence 24-hour rhythms^17,18^. Our previous report of temporal gene expression in the same depot of human adipose tissue^14^ assessed daily variation of clock gene expression in entrained (‘diurnal’) conditions. Nonetheless the phase of canonical clock gene expression is highly conserved between our two studies. Our previously reported diurnal rhythms described a peak of *BMAL1*/*ARNTL* expression in the late evening with *PERs*, *CRY2*, and *REVERBα*/*NR1D1* peaking around late morning^14^. In the current study, *BMAL1*/*ARNTL* exhibited peak expression in the late biological evening, with the other clock genes peaking in mid-late biological morning. Our data add to existing knowledge of adipose rhythms^20^ and indicate that robust circadian rhythmicity exists in human adipose tissue *in vivo* and is associated with fundamental metabolic processes.

Assessment of rhythmicity in the transcriptome was performed using methods that allow the characterization of an expression profile based on its phase, amplitude and period, similar to the methods previously used for analysis of the circadian transcriptome in human whole blood^12,13^. The estimate that ≈2% of the adipose transcriptome exhibits circadian rhythms is lower than the value estimated in previous studies, as reviewed elsewhere^11^ but still represents many hundreds of circadian genes. The lower estimate of circadian gene prevalence may be a feature of the tissue but may also reflect protocol design, technological platforms, and the analytical algorithms used to identify rhythmic signals, as discussed previously^11^. The cohort size and sampling resolution within our study is also lower than that recently recommended for genome-wide circadian analysis^21^, but is a necessary ethical and practical limitation when taking serial human biopsies. Our circadian genes separated into two clearly defined groups with a bimodal distribution of peak expression. A similar bimodal distribution has been reported in the circadian transcriptome of human blood^12,13^ and diurnal transcriptome of human skeletal muscle^16^ indicating that it is a common feature of 24-hour organisation of molecular processes in humans.

The results of our transcriptome analysis were first interpreted using Gene Ontology (GO) enrichment analysis. Biological processes in GO annotation highlight the overall biological purpose of a gene’s product whilst molecular function relates to the specific function of a gene product^22^. For the morning-peaking genes, GO enrichment revealed processes relating to circadian rhythms, as most of the clock genes had peak expression in the morning. In addition, GO analysis revealed prominent roles of morning-peaking genes related to gene expression (e.g. transcription factor and nucleic acid binding; histone deacetylation) and metabolism of nitrogen-containing compounds. A study examining the subcutaneous human adipose tissue transcriptome in biopsies taken over a 12-hour period also found that the function of morning genes was enriched for transcription and translation^23^. Evening-peaking genes saw GO enrichment in multiple metabolic processes including organic acid metabolism. Enriched molecular functions included oxidoreductase activity and lytic activity. Consistent with this finding, mouse white adipose rhythms are closely involved in lipolysis^24^ indicating a conserved circadian involvement in catalytic functions.

Comparison of our data with published circadian transcriptome datasets yielded a relatively small number of common circadian genes. Notwithstanding the core clock genes, circadian transcriptome data are typically specific to the relevant tissue(s) in question^10,11^. Comparing circadian genes in human adipose tissue from the current study with those in human blood is potentially complicated by the fact that our study only involved male participants, whereas the studies of human blood included mixed male and female participants. Nonetheless, the non-clock genes common to all three studies were *BMS1*, *CLEC18B*, *CLUAP1*, *COTL1*, *MPZL1*, *PAIP2B* and *RPL22*. Products of these genes have very different functions: BMS1 is a likely ribosome biogenesis factor (by similarity), CLEC18B binds polysaccharides^25^, CLUAP1 functions in cilia biogenesis and hedgehog signalling^26^, COTL1 helps to regulate the actin skeleton^27^, MPZL1 is involved in signal transduction^28^, whereas PAIP2B and RPL22 are involved in translation^29,30^. Circadian genes that were common to human adipose tissue and at least one of the blood datasets were involved in fundamental processes for cell function; namely circadian rhythm regulation, metabolic processes and transcription and translation.

The low overlap between circadian transcriptomes in human and mouse adipose tissue could derive from species differences as well as different physiological properties of the adipose depots^31^. In addition to the clock genes, conserved rhythmicity was observed in *Cpsf1*, *Gstt2*, *Hlf*, *Rev1*, *Plce1, Nqo2, Rev1* and *Timm10*. These genes have roles like those seen in the GO biological enrichment. CPSF1 is involved in processing the 3′ end of pre-mRNA^32^; HLF is a transcription factor^33^; GSTT2 catalyses glutathione conjugation to both hydrophilic and hydrophobic compounds^34^; and REV1 is involved in DNA polymerase-mediated repair of DNA^35^. Similarly, genes in both adipose depots were involved with metabolism; PLCE1 catalyses PIP_2_ into secondary messenger molecules IP3 and DAG^36^; NQO2 reduces quinone substrates^37^ and TIMM10 inserts hydrophobic proteins into the mitochondrial inner membrane^38^.

We finally assessed the functional consequence of our transcriptomic data through molecular interactions and metabolic pathway activity. The inferred/known molecular interaction networks supported the GO findings, highlighting networks involved in circadian rhythms and key aspects of cellular metabolism. In addition, STRING analysis revealed clusters regulating other core aspects of cell biochemistry, such as mRNA processing/splicing, protein trafficking and Golgi function. Application of our transcriptomic data to human metabolic network models further supported key roles of adipose circadian rhythms in: fatty acid metabolism; nucleic acid metabolism; and other fundamental aspects of cell function, such as the citric acid cycle. These novel findings indicate the association of circadian processes with specific and critical metabolic pathways in cellular metabolism of human white adipose tissue.

Our study has some limitations. As noted above, we only studied male participants. Previous research has demonstrated gender differences in circadian rhythmicity^39,40^ and white adipose physiology varies between men and women^41^. It will therefore be important to compare rhythms in a wider population in future work. The sample size reported in this manuscript is quite small, although comparable to many controlled laboratory human studies of this nature. Sample size is limited by the cost and ethical considerations of running such complex human studies. However, it must be emphasised that an important advantage of our study is that we were able to obtain repeated samples from each participant.

## Conclusions

Molecular analysis of serial adipose tissue biopsies, taken under highly controlled conditions and coupled with in-depth bioinformatic analysis, has revealed the importance of circadian biology on a key human metabolic tissue. The most common rhythmic processes in human subcutaneous white adipose are those linked to fatty acid metabolism. However, rhythmicity was also observed in other fundamental cell processes, e.g. transcription and translation, nucleic acid metabolism and the citric acid cycle. The circadian timing system thus has an intimate relationship with many core aspects of human physiology and pathways relevant to therapeutics.

## Methods

### Participants

The study received a favourable ethical opinion from the University of Surrey Ethics Committee and was conducted in accordance with the guidelines laid down in the Declaration of Helsinki. Written, informed consent was obtained prior to any study procedures being performed. We recruited seven participants, who were male, aged 18–30 years with body mass index (BMI) ≥ 19 kg/m^2^ and ≤30 kg/m^2^ and fat mass > 14%. These participants were part of a cohort recruited for a previously published study^18^. They had no medical history that indicated a sleep or metabolic disorder and had not undertaken shift work within six months or crossed more than two time zones within one month of the study. Participants (Table 3) completed a set of validated sleep and chronotype questionnaires and were required to meet the following inclusion criteria: Pittsburgh Sleep Quality Index (score ≤ 5), Epworth Sleepiness Scale (score < 10), Horne-Östberg diurnal preference questionnaire (score between but not including 30–70, indicating that they are not extreme morning or evening types). Their habitual bed time was between 22:00 and 01:00 and wake time between 06:00–09:00 for 5 nights a week; habitual sleep duration was 7–9 hours per night.

**Table 3:** Participant demographics.

*Physiological Variables*	Mean ± S.D.
Age (years)	22.9 ± 3.4
Body Mass Index (kg/m^2^)	23.9 ± 2.4
Body Fat (%)	16.8 ± 6.0
***Questionnaire Data***	**Mean ± S.D**.
Munich Chronotype	
Sleep Time (decimal hr)	23.21 ± 0.39
Wake Time (decimal hr)	07.88 ± 0.53
Horne-Östberg score	50.0 ± 7.8
Pittsburgh Sleep Quality Index	2.9 ± 1.2
Beck Depression Inventory	1.0 ± 1.2
Epworth Sleepiness Scale	5.1 ± 1.3
***Ethnicity***	***N***
White	6
African	1

All participants were medically assessed (electrocardiogram, blood pressure, heart rate, oral temperature and respiration rate) at the Surrey Clinical Research Centre (CRC) as part of a screening visit. They were required to test negative for drugs of abuse such as opiates, alcohol and cotinine, a metabolite of nicotine. A full blood biochemistry screen was conducted to ensure good health and medication records were reviewed.

### Study design

Ten days prior to the study session, participants were instructed to select and maintain a bed time (between 22:00 and 01:00) and wake time (between 06:00 and 09:00) that closely matched their habitual sleep-wake time, with a time-in-bed duration of at least 8 hours. If required, a nap was allowed within a 4-hour window, with the middle of the nap window being 12 hours after the midpoint of sleep. Additionally, participants were required to obtain 15 minutes of unobstructed natural light exposure within 1.5 hours of wake. Compliance was monitored using two L-actiwatches (Cambridge Neurotechnology Ltd, Cambridge, UK). One actiwatch was worn on the non-dominant wrist for activity analysis, whereas the other was worn around the neck for analysis of light exposure. Participants maintained a sleep diary and called a time-stamped voice mail within 10 minutes of the selected bed and wake times.

Seven days prior to the study, participants were asked to restrict the time they ate their meals to the following: breakfast 30 minutes after wake, lunch 5.5 hours after wake and dinner 10.5 hours after wake. Caffeine was restricted to no more than 100 mg in the first three hours of waking, and a maximum of 4–5 units of alcohol was allowed per day. Seventy-two hours prior to the laboratory study, participants were given food by the study team to resemble the food they would consume during the study. They were required to maintain the meal schedule detailed above and, in addition, they were not allowed to consume any caffeine or alcohol and were asked to refrain from any heavy exercise during those 72-hours.

Participants entered the Surrey CRC, received a standard meal and went to bed in individual sleep rooms at the same time as they had selected during the pre-study protocol. Over the next 3 days, participants continued their same sleep-wake and meal schedule. All meals were isocaloric with the same macronutrient content (55% carbohydrate, 15% protein and 30% fat) and eaten in individual rooms. Meals were calorie adjusted to meet the individual energy requirements of the participant using the Schofield equation^42^. Participants could move around when not eating, but no strenuous activity was allowed. Over laboratory days 4 and 5, participants underwent a 37-hour constant routine. Sampling during the constant routine began five hours into the constant conditions to eliminate any run-in effects and consisted of hourly blood sampling and five six-hourly subcutaneous adipose tissue biopsies from the upper gluteal region, which is known to be metabolically active^43^. Biopsy tissue (≈200 mg per biopsy) was snap frozen in liquid nitrogen and stored at −80 °C until RNA extraction. Blood was collected into tubes containing lithium heparin anticoagulant, inverted 10 times, cooled, and centrifuged (1,620 *g*, 10 mins, 4 °C) within 30 minutes of collection. Plasma was then collected and stored at −20 °C ready for analysis.

### Plasma melatonin analysis

All plasma melatonin determinations were conducted by Stockgrand Ltd (University of Surrey) using a tritium-based assay described elsewhere^44^. Dim light melatonin onset (DLMO) was calculated using the 25% method to permit alignment of gene expression to an endogenous circadian phase marker^45^.

### Microarray analysis of biopsy tissue

RNA*later*-ICE solution (Thermo Fisher Scientific) was pre-chilled at −80 °C and 10 µl per mg of tissue was added to the frozen adipose tissue. The tissue was then kept for a minimum of 16 hours at −20 °C. RNA was extracted from approx 30 mg of the thawed adipose tissue using the RNeasy mini kit (Qiagen, Hilden, Germany) according to the manufacturer’s protocol. The RNA was then assessed for quantity and quality using the NanoDrop 2000 spectrophotometer (LabTech International) and Agilent 2100 Bioanalyzer (Agilent Technologies) with the RNA 6000 Nano Kit. Samples had an average RNA Integrity Number (RIN) of 7.3 ± 0.72 (mean ± SEM). RNA was labelled and amplified to produce cRNA using the one-colour low input quick amp labelling kit (Agilent Technologies). Hybridisation of cRNA to arrays (Agilent, human whole genome custom microarrays 4 × 44 k as described in the Gene Expression Omnibus, GEO^46,47^; platform ID: GPL15331) was performed for 17 hours at 65 °C following Agilent Technologies instructions. Once slides had been washed and scanned using an Agilent microarray scanner, data were extracted using Agilent Feature Extraction Software (Agilent [version 11.5.1.1]).

Individual samples were filtered based on the AgilentQC metrics provided by Agilent Feature Extraction software. Only one sample was excluded based on our criteria that a sample should have a median coefficient of variation (CV) of less than 10% in spike-ins or in non-control replicated probes. For the remaining samples log_2_ mRNA abundance values were quantile-normalized across all arrays using the R Bioconductor package limma^48^. Non-control replicated probes, along with their corresponding flags were averaged. Probes with more than 66% of samples flagged by Agilent FE software were not considered. The microarray dataset is accessible from GEO via accession number GSE87761.

Circadian alignment of subjects was conducted by referencing each participant’s sampling times to the participant’s melatonin onset (DLMO value). A single component cosinor, with added linear trend and assuming 24 h period, was fitted to the mRNA abundance profile of each microarray probe by a linear mixed model with subjects as random effects using the lme4 package from R^49^. A circadian oscillation was considered if the mixed model fit had an R^2^ value higher than 0.8 and the 95% confidence interval (CI) of the calculated amplitude did not include the zero value. The false discovery rate (FDR) was calculated as the expected proportion of the circadian probes that are classed as circadian after sample labels within each subject had been randomly permuted. A total of 100 random permutations were done for each probe independently and FDR was <10%.

Note that most alternative methods for determining rhythmicity make assumptions that are not compatible with our data. They assume replicated time points and evenly sampling points, i.e. all data samples come from different participants, there are several measurements for the same time point and sampling is performed at regular, fixed intervals. Our dataset has replicated time-series instead of replicated time points. Each participant provided data for all time points and although the sampling interval is similar across participants, the sampling time points are unique as they are adjusted to each participant’s melatonin profile.

Probes that had been identified as rhythmic underwent unsupervised clustering^12,13,50^ using a circular self-organising map^50^. Three clusters were identified as the optimal partition based on the Bayesian Information Criterion (BIC)^51^. For further downstream analysis/interpretation, two of these three clusters exhibiting peak expression within 5-hours of DLMO were analysed together and defined as evening-peaking. The third cluster was defined as morning-peaking.

Gene ontology (GO) enrichment analysis was conducted using Metacore software (Version 6.33, Build 69110). GO biological processes and molecular functions that had an enrichment p value and a corresponding FDR of less than 0.05 were considered to be significantly enriched^52^. Molecular interactions were identified/predicted using the online search tool STRING (www.string-db.org; V 10.5)^53^. The gene symbols associated with each transcript belonging to a cluster were entered as a multiple protein search. Default settings were used to identify/predict interactions with a minimum interaction (confidence) score of 0.4, corresponding to medium level of confidence.

### Comparison of rhythmic genes with other circadian datasets

Genes associated with circadian transcripts were compared to rhythmically expressed genes identified by transcriptome analyses of mouse organs^10^, human whole blood^12,13^ and Olive Baboon^19^. Ensembl Biomart^54^ was used to identify homologous genes between organisms and cross-referenced with the gene symbols reported by each of the transcriptome studies. Expression profiles and peak times for rhythmic genes in mouse white adipose tissue were extracted from CircaDB^55^.

### Metabolic network analysis

To predict the activity of metabolic reactions within the human system the ‘Fast iMAT’ algorithm of MUFINS^56^, a variant of the iMAT algorithm^57,58^, was used. Under a constraint-based modelling framework, iMAT approaches aim to maximise the congruency between functional -omic data and the activity of metabolic reactions in a metabolic model. Here, for each of the five time points assessed, we conducted Fast iMAT analysis using the average (across participants) log_2_ transcriptome sample and the human genome-scale metabolic network model, Recon2^59^, as input. Probes targeting transcripts of the same gene were averaged, producing a single mRNA abundance value for a given gene at a specific sampling time point. Averaged mRNA abundance values were discretised into three levels (−1, 0, 1) to represent three activity states (inactive, neutral, and active, respectively) for that sampling time point, using the percentile thresholds of ≤20% (state −1), >20% and ≤80% (state 0), and >80% (state 1). All exchange reactions (reactions that control the uptake/excretion of metabolites) in the Recon2 model were unbounded. Subsequent outputs of Fast iMAT included the predicted activity state (−1, 0, 1) for each of the 10,770 reactions within the Recon2 model for each time point. Thus, for each reaction within the metabolic model we obtained a predicted metabolic activity profile across the circadian cycle. Metabolic reactions for which we obtained an activity profile exhibiting at least one change in state were further analysed. The fit (R^2^) of each metabolic activity profile to a sinusoidal function was assessed and a threshold of R^2^ ≥ 0.8 was used to identify predicted rhythmic metabolic activity profiles.

Metabolic reactions identified as rhythmic (predicted metabolic activity profile R^2^ ≥ 0.8) were mapped to the genes annotated as responsible for carrying out these reactions in the Recon2 model. The mapped genes were subsequently mapped to human KEGG pathway maps^60–62^ using the R package PathView^63^ for visualisation.

## Supplementary information

Supplementary information

Supplementary Dataset 1

Supplementary Dataset 2

## Data Availability

The microarray dataset is accessible from GEO via accession number GSE87761.
