# Supplementary material for: Circadian regulation in human white adipose tissue revealed by transcriptome and metabolic network analysis
Source: Sci Rep. 2019 Feb 25;9:2641. doi: 10.1038/s41598-019-39668-3 (PMC6389935; doi:10.1038/s41598-019-39668-3)
Supplement: Supplementary file 1 — Supplementary information [file 41598_2019_39668_MOESM1_ESM.pdf]

Supplementary Information:

Circadian regulation in human white adipose tissue revealed by transcriptome and metabolic network analysis

Skevoulla Christou [1], Sophie M T Wehrens [1], Cheryl Isherwood [1,2], Carla S Möller-Levet [3], Huihai Wu [3], Victoria L Revell [1], Giselda Bucca [1,4], Debra J Skene [1], Emma E Laing [1], Simon N Archer [1,5], Jonathan D Johnston [1,5]

[1] Faculty of Health and Medical Sciences, University of Surrey, UK
[2] Present address: Department of Medicine, Brigham and Women's Hospital, Boston, USA
[3] Bioinformatics Facility, Faculty of Health and Medical Sciences, University of Surrey, UK
[4] Present address: School of Pharmacy and Biomolecular Sciences, University of Brighton, UK
[5] Shared senior authorship

Corresponding author: Dr Jonathan D Johnston
Faculty of Health and Medical Sciences
University of Surrey
Guildford
Surrey GU2 7XH
UK
email: j.johnston@surrey.ac.uk
tel: 44 (0) 1483 686470

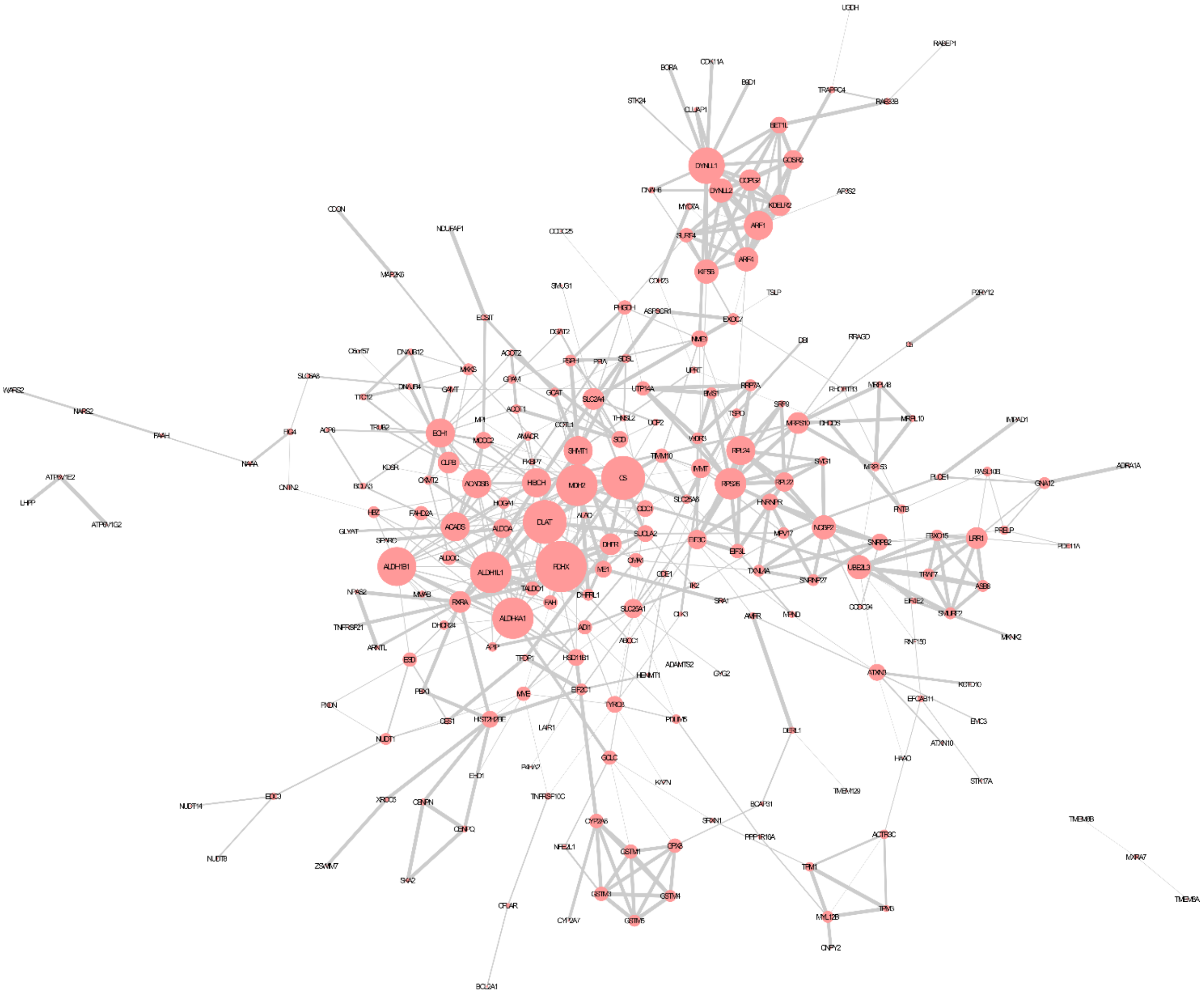

Supplementary Figure S1: Molecular interaction network associated with evening-peaking genes. Molecular interaction network for genes associated with transcripts that peak during the evening were computed using the STRING online database. Node size reflects the number of direct connections a molecule has within the network. Thickness of lines (edges) connecting nodes represents strength of evidence (confidence) supporting each connection, as provided by STRING.

Supplementary Table S1 – Circadian transcripts in human white adipose tissue

	Probe ID	R^2	Amplitude ± SE	Peak Time (Relative to the DLMO)	Morning or Evening Peaking
ERAP2	A_23_P30243	0.9930	0.1 ± 0.041	11.53	Morning
PSPH	A_32_P78816	0.9903	0.103 ± 0.045	-0.06	Evening
STON1-GTF2A1L	A_23_P79488	0.9873	0.266 ± 0.075	-3.69	Evening
LOC401233	A_32_P135890	0.9842	0.124 ± 0.061	0.17	Evening
RPS26	A_24_P638294	0.9803	0.057 ± 0.027	-2.27	Evening
IMPAD1	A_24_P101200	0.9795	0.19 ± 0.067	-4.77	Evening
A_24_P263443	A_24_P263443	0.9773	0.071 ± 0.031	0.05	Evening
RPS26	A_24_P289404	0.9754	0.094 ± 0.034	-0.90	Evening
LOC389607	A_24_P660811	0.9745	0.169 ± 0.082	17.78	Evening
GSTM3	A_23_P12343	0.9697	0.06 ± 0.026	-3.36	Evening
LOC283788	A_24_P497437	0.9692	0.259 ± 0.059	8.97	Morning
A_23_P252125	A_23_P252125	0.9672	0.141 ± 0.054	4.48	Morning
LOC283481	A_32_P79483	0.9648	0.204 ± 0.058	-3.09	Evening
STAT6	A_23_P47879	0.9633	0.1 ± 0.041	6.46	Morning
BC073929	A_32_P199263	0.9607	0.22 ± 0.078	9.99	Morning
A_32_P168431	A_32_P168431	0.9606	0.09 ± 0.034	-3.18	Evening
RABEP1	A_23_P78158	0.9583	0.183 ± 0.053	-4.45	Evening
ALDOC	A_23_P78108	0.9582	0.19 ± 0.066	-3.03	Evening
BC035147	A_32_P507580	0.9576	0.169 ± 0.053	17.85	Evening
ENST00000427651	A_24_P49597	0.9574	0.166 ± 0.068	-1.89	Evening
LRRC36	A_23_P118042	0.9571	0.255 ± 0.111	3.68	Morning
THNSL2	A_23_P259207	0.9561	0.18 ± 0.047	-3.92	Evening
AKR7A2	A_23_P115356	0.9545	0.146 ± 0.054	-0.50	Evening
AKR7L	A_23_P320304	0.9539	0.119 ± 0.047	-1.66	Evening
HMBOX1	A_23_P134684	0.9521	0.199 ± 0.05	9.55	Morning
MAPK8IP1	A_24_P409595	0.9513	0.168 ± 0.084	-1.52	Evening
A_24_P161494	A_24_P161494	0.9493	0.185 ± 0.087	0.22	Evening
MTHFSD	A_32_P149111	0.9483	0.167 ± 0.049	7.32	Morning
GSTM3	A_24_P914434	0.9481	0.138 ± 0.041	-2.06	Evening
GLIPR1L2	A_24_P381499	0.9480	0.198 ± 0.073	-0.57	Evening
CGREF1	A_23_P403445	0.9476	0.136 ± 0.068	-3.69	Evening
MYOM2	A_23_P258912	0.9468	0.176 ± 0.064	-2.11	Evening
PSPH	A_23_P251984	0.9444	0.125 ± 0.047	-0.39	Evening
A_32_P103966	A_32_P103966	0.9430	0.397 ± 0.152	11.03	Morning
CYP4V2	A_32_P23838	0.9428	0.077 ± 0.036	15.02	Evening
A_32_P151747	A_32_P151747	0.9427	0.224 ± 0.092	-1.95	Evening
GSTM4	A_23_P217917	0.9416	0.219 ± 0.06	-3.03	Evening
MRPL42P5	A_23_P398491	0.9413	0.203 ± 0.054	8.77	Morning
A_32_P168727	A_32_P168727	0.9394	0.143 ± 0.065	-2.19	Evening

NCBP2	A_24_P157424	0.9371	0.179 ± 0.046	-1.80	Evening
BG777521	A_24_P647163	0.9370	0.133 ± 0.048	-1.47	Evening
ARL17P1	A_24_P104538	0.9348	0.082 ± 0.03	5.64	Morning
NAAA	A_23_P155666	0.9347	0.135 ± 0.057	-0.77	Evening
AMFR	A_23_P141005	0.9333	0.096 ± 0.042	17.91	Evening
CKMT2	A_23_P144778	0.9332	0.175 ± 0.07	-5.86	Evening
PAIP2B	A_23_P301053	0.9304	0.385 ± 0.132	-1.79	Evening
BTN3A3	A_24_P311917	0.9300	0.206 ± 0.049	-2.30	Evening
CLSTN2	A_23_P212608	0.9289	0.154 ± 0.056	-4.17	Evening
PHGDH	A_23_P85783	0.9280	0.223 ± 0.101	0.41	Evening
NUDT14	A_23_P414978	0.9272	0.172 ± 0.062	-2.99	Evening
BG719660	A_24_P3016	0.9271	0.194 ± 0.095	-1.90	Evening
TALDO1	A_32_P28872	0.9261	0.142 ± 0.03	-3.51	Evening
CR622844	A_24_P579780	0.9252	0.118 ± 0.049	9.33	Morning
BC034271	A_23_P319970	0.9248	0.184 ± 0.081	-2.34	Evening
GTF3C5	A_23_P112406	0.9247	0.066 ± 0.024	4.28	Morning
PPIL3	A_23_P28213	0.9244	0.082 ± 0.034	8.18	Morning
PCM1	A_24_P555510	0.9236	0.107 ± 0.043	4.27	Morning
NR1D1	A_23_P420873	0.9229	1.863 ± 0.106	6.08	Morning
FLJ30064	A_24_P76512	0.9216	0.11 ± 0.047	9.92	Morning
LOC340947	A_24_P177653	0.9214	0.32 ± 0.104	-1.10	Evening
SFTPD	A_32_P107617	0.9213	0.185 ± 0.086	-1.10	Evening
TMEM8A	A_23_P140907	0.9212	0.162 ± 0.037	-4.41	Evening
CR603845	A_32_P32722	0.9211	0.131 ± 0.039	-1.47	Evening
FAM149B1	A_24_P230907	0.9211	0.102 ± 0.042	-1.94	Evening
LOC730091	A_32_P224327	0.9210	0.117 ± 0.054	5.46	Morning
ICAM3	A_23_P164691	0.9209	0.155 ± 0.048	-2.87	Evening
TTC3	A_32_P165330	0.9204	0.205 ± 0.048	-2.00	Evening
LOC283788	A_32_P80089	0.9202	0.221 ± 0.068	9.41	Morning
CES1	A_23_P206733	0.9200	0.202 ± 0.078	-4.91	Evening
PPIAL4A	A_24_P144499	0.9200	0.21 ± 0.076	-1.53	Evening
BC014971	A_23_P140884	0.9196	0.261 ± 0.08	7.53	Morning
LOC284412	A_24_P375691	0.9193	0.159 ± 0.068	9.77	Morning
BC014871	A_24_P102406	0.9192	0.348 ± 0.118	9.69	Morning
BC006271	A_24_P316381	0.9182	0.201 ± 0.087	10.84	Morning
ZSWIM7	A_23_P164421	0.9175	0.119 ± 0.052	-3.31	Evening
GTF2A1L	A_23_P79496	0.9172	0.59 ± 0.135	-4.29	Evening
KCNK15	A_23_P210451	0.9171	0.262 ± 0.105	-5.44	Evening
SCD	A_24_P626920	0.9170	0.333 ± 0.122	16.72	Evening
TOLLIP	A_24_P287189	0.9166	0.352 ± 0.065	-3.04	Evening
LOC100132288	A_24_P306443	0.9161	0.213 ± 0.092	-5.00	Evening
BQ948285	A_32_P205478	0.9152	0.142 ± 0.036	7.73	Morning
NR1D2	A_24_P943472	0.9147	0.805 ± 0.048	7.64	Morning
RPL23AP7	A_24_P307025	0.9139	0.197 ± 0.038	-0.92	Evening
A_32_P188127	A_32_P188127	0.9130	0.364 ± 0.109	-0.30	Evening
HSP90AA4P	A_24_P118142	0.9119	0.272 ± 0.067	-0.90	Evening

DDT	A_23_P17769	0.9116	0.082 ± 0.035	-2.68	Evening
FGFBP3	A_23_P417994	0.9113	0.137 ± 0.033	8.46	Morning
A_32_P93144	A_32_P93144	0.9111	0.339 ± 0.105	12.59	Morning
GSTT2	A_23_P109427	0.9106	0.229 ± 0.066	8.86	Morning
ERC1	A_24_P162293	0.9106	0.085 ± 0.043	-2.03	Evening
SLC6A8	A_23_P159937	0.9100	0.197 ± 0.047	-3.08	Evening
AL546498	A_32_P186725	0.9099	0.123 ± 0.044	-0.78	Evening
DNAH6	A_23_P324605	0.9097	0.357 ± 0.137	-0.66	Evening
NME1	A_23_P152804	0.9096	0.097 ± 0.041	-0.84	Evening
DHFR	A_24_P320284	0.9096	0.225 ± 0.054	-2.62	Evening
KIAA0020	A_23_P20683	0.9092	0.125 ± 0.027	5.83	Morning
C21orf81	A_23_P392529	0.9084	0.33 ± 0.148	9.65	Morning
tcag7.873	A_24_P75456	0.9081	0.422 ± 0.117	-0.69	Evening
NQO2	A_23_P58953	0.9073	0.19 ± 0.072	-2.08	Evening
A_32_P16931	A_32_P16931	0.9073	0.161 ± 0.066	-2.54	Evening
CYP20A1	A_24_P394047	0.9072	0.063 ± 0.032	-1.58	Evening
SERGEF	A_23_P139207	0.9064	0.142 ± 0.066	0.05	Evening
A_24_P914062	A_24_P914062	0.9064	0.19 ± 0.092	11.99	Morning
A_24_P641673	A_24_P641673	0.9060	0.329 ± 0.079	-1.82	Evening
AF126109	A_32_P162726	0.9051	0.057 ± 0.027	12.69	Morning
TIMM10	A_23_P64343	0.9051	0.135 ± 0.052	-1.17	Evening
A_32_P219377	A_32_P219377	0.9049	0.113 ± 0.049	-4.59	Evening
GAMT	A_24_P19228	0.9046	0.156 ± 0.048	-2.61	Evening
ESPNL	A_24_P170983	0.9045	0.207 ± 0.08	9.13	Morning
RAB33B	A_24_P268917	0.9038	0.153 ± 0.057	-3.63	Evening
XRCC5	A_24_P345498	0.9035	0.161 ± 0.044	-2.26	Evening
SNORD123	A_24_P639679	0.9033	0.177 ± 0.079	-1.45	Evening
AK055501	A_24_P538567	0.9031	0.145 ± 0.051	-1.97	Evening
USP36	A_23_P352957	0.9027	0.283 ± 0.054	8.18	Morning
A_24_P594683	A_24_P594683	0.9025	0.138 ± 0.048	-3.42	Evening
TRAF7	A_24_P53150	0.9022	0.176 ± 0.056	-3.76	Evening
UPRT	A_24_P185986	0.9019	0.051 ± 0.026	-5.04	Evening
GSTM1	A_23_P115407	0.9016	0.211 ± 0.047	-3.23	Evening
NR1D1	A_24_P250227	0.9016	1.683 ± 0.107	6.04	Morning
A_32_P15898	A_32_P15898	0.9014	0.202 ± 0.087	-1.46	Evening
DBI	A_23_P79199	0.9014	0.117 ± 0.054	-1.58	Evening
FAM86C	A_24_P218757	0.9009	0.108 ± 0.042	-1.34	Evening
A_32_P194563	A_32_P194563	0.8997	0.083 ± 0.037	9.45	Morning
DHFR	A_24_P343095	0.8991	0.224 ± 0.069	-2.37	Evening
FRG1B	A_24_P417526	0.8987	0.084 ± 0.041	8.82	Morning
DHRS1	A_23_P48747	0.8986	0.088 ± 0.042	2.82	Morning
CN294989	A_24_P733308	0.8983	0.561 ± 0.137	8.26	Morning
AK126976	A_32_P19966	0.8982	0.544 ± 0.122	12.35	Morning
A_24_P401051	A_24_P401051	0.8979	0.108 ± 0.04	16.10	Evening
CARD8	A_24_P14260	0.8972	0.136 ± 0.064	13.19	Morning
ZFP90	A_24_P176404	0.8970	0.167 ± 0.034	8.97	Morning

C15orf57	A_32_P98975	0.8969	0.137 ± 0.037	-5.82	Evening
C1orf59	A_23_P309361	0.8969	0.167 ± 0.028	-0.27	Evening
MUC20	A_23_P92222	0.8968	0.39 ± 0.094	9.08	Morning
A_24_P680548	A_24_P680548	0.8966	0.338 ± 0.072	-1.94	Evening
CLPB	A_23_P75978	0.8964	0.171 ± 0.049	-3.63	Evening
ARSA	A_23_P255436	0.8953	0.136 ± 0.035	-2.73	Evening
LOC100192204	A_24_P708363	0.8949	0.18 ± 0.08	-1.30	Evening
PRUNE2	A_23_P406025	0.8947	0.26 ± 0.105	-3.58	Evening
ANKRD20A2	A_32_P168326	0.8946	0.412 ± 0.084	10.10	Morning
CLIC6	A_23_P385067	0.8938	0.193 ± 0.087	-4.68	Evening
CYP2A6	A_23_P16122	0.8931	0.249 ± 0.094	-1.85	Evening
BX116163	A_32_P89997	0.8931	0.248 ± 0.089	0.69	Evening
HEXIM2	A_23_P377214	0.8930	0.194 ± 0.029	-3.34	Evening
MGC70870	A_32_P221256	0.8923	0.119 ± 0.05	0.56	Evening
SPATA18	A_23_P407112	0.8919	0.266 ± 0.083	-0.67	Evening
A_24_P324074	A_24_P324074	0.8918	0.29 ± 0.087	-1.03	Evening
AFARP1	A_23_P316812	0.8917	0.242 ± 0.07	-1.44	Evening
ADI1	A_23_P148162	0.8917	0.187 ± 0.057	-2.20	Evening
CYP20A1	A_23_P56894	0.8912	0.122 ± 0.037	-0.52	Evening
FBXO15	A_23_P342709	0.8912	0.237 ± 0.064	-2.37	Evening
MFF	A_23_P92994	0.8911	0.11 ± 0.046	-1.46	Evening
FN3KRP	A_23_P77813	0.8907	0.084 ± 0.032	-4.58	Evening
PTN	A_23_P303087	0.8901	0.371 ± 0.1	5.51	Morning
HIBCH	A_23_P154345	0.8897	0.095 ± 0.032	1.17	Evening
ADI1	A_32_P52911	0.8896	0.181 ± 0.057	-2.62	Evening
ARL17P1	A_24_P579826	0.8895	0.137 ± 0.054	6.88	Morning
SLC6A10P	A_24_P15621	0.8895	0.152 ± 0.035	17.61	Evening
CR620532	A_24_P354257	0.8892	0.11 ± 0.048	1.61	Evening
RBM17	A_23_P35650	0.8889	0.167 ± 0.049	9.77	Morning
LOC151174	A_24_P469746	0.8886	0.236 ± 0.114	-1.94	Evening
ANKRD20A2	A_24_P868583	0.8886	0.34 ± 0.075	9.91	Morning
ADI1	A_23_P148194	0.8874	0.161 ± 0.037	-2.31	Evening
HBZ	A_23_P3651	0.8870	0.308 ± 0.102	-2.12	Evening
ALAD	A_23_P324278	0.8870	0.115 ± 0.045	-2.89	Evening
MPZL1	A_23_P11874	0.8864	0.202 ± 0.036	-3.86	Evening
A_24_P375132	A_24_P375132	0.8864	0.128 ± 0.048	-1.67	Evening
MR1	A_23_P201837	0.8858	0.089 ± 0.041	-2.23	Evening
GGNBP2	A_24_P67976	0.8857	0.093 ± 0.021	2.90	Morning
GPAM	A_24_P227069	0.8855	0.224 ± 0.079	15.81	Evening
A_32_P101019	A_32_P101019	0.8854	0.272 ± 0.12	9.90	Morning
APIP	A_23_P2066	0.8851	0.23 ± 0.052	-1.05	Evening
SKA2	A_32_P164522	0.8851	0.082 ± 0.029	-4.79	Evening
ESD	A_23_P87964	0.8850	0.163 ± 0.04	-1.70	Evening
RFFL	A_23_P164089	0.8848	0.155 ± 0.038	-1.85	Evening
A_23_P212626	A_23_P212626	0.8846	0.376 ± 0.091	8.21	Morning
A_32_P55934	A_32_P55934	0.8846	0.141 ± 0.066	1.45	Evening

RP3-398D13.1	A_32_P178758	0.8846	0.265 ± 0.134	8.40	Morning
FGFBP2	A_23_P41528	0.8842	0.363 ± 0.111	-2.63	Evening
MKNK2	A_23_P142304	0.8839	0.21 ± 0.056	-2.22	Evening
LOC100130794	A_24_P76078	0.8839	0.316 ± 0.115	-0.52	Evening
DHFRL1	A_23_P139312	0.8835	0.175 ± 0.04	-3.21	Evening
TMEM8B	A_23_P216522	0.8833	0.086 ± 0.026	2.61	Evening
NCOA4	A_23_P86421	0.8831	0.086 ± 0.028	17.56	Evening
PARP4	A_23_P117175	0.8831	0.106 ± 0.047	-1.89	Evening
CRY2	CPID_285/A_23_P388027	0.8829	0.374 ± 0.037	9.66	Morning
A_24_P238996	A_24_P238996	0.8826	0.235 ± 0.056	3.32	Morning
SURF4	A_24_P89971	0.8821	0.177 ± 0.041	-3.20	Evening
BG618521	A_32_P8653	0.8818	0.441 ± 0.093	-3.82	Evening
AK124841	A_24_P778844	0.8816	0.218 ± 0.086	17.28	Evening
ZBTB7B	A_23_P11729	0.8812	0.146 ± 0.047	-1.84	Evening
PCYT2	A_24_P404245	0.8812	0.206 ± 0.057	-2.52	Evening
SMA5	A_23_P121869	0.8808	0.206 ± 0.057	7.87	Morning
TTC23	A_23_P140563	0.8807	0.095 ± 0.048	-0.74	Evening
C5	A_23_P71855	0.8798	0.148 ± 0.059	-0.57	Evening
RPS26	A_23_P116694	0.8797	0.162 ± 0.056	-3.88	Evening
SLC25A1	A_23_P120776	0.8793	0.179 ± 0.052	-3.05	Evening
C9orf110	A_32_P99347	0.8792	0.155 ± 0.077	-4.54	Evening
TMEM111	A_32_P194095	0.8792	0.067 ± 0.028	15.45	Evening
RHBDF2	A_23_P329870	0.8791	0.318 ± 0.078	12.29	Morning
TTC32	A_23_P119857	0.8790	0.23 ± 0.055	6.57	Morning
TSPO	A_24_P134526	0.8788	0.109 ± 0.051	-1.88	Evening
C17orf101	A_24_P940790	0.8785	0.137 ± 0.045	-3.47	Evening
A_32_P77252	A_32_P77252	0.8785	0.363 ± 0.094	7.26	Morning
A_32_P95894	A_32_P95894	0.8783	0.158 ± 0.05	1.54	Evening
A_24_P67618	A_24_P67618	0.8781	0.315 ± 0.054	-2.58	Evening
PDPK1	A_24_P222599	0.8775	0.087 ± 0.039	7.75	Morning
NBPF1	A_24_P566932	0.8774	0.376 ± 0.07	-2.58	Evening
DHX58	A_23_P38346	0.8773	0.194 ± 0.037	9.24	Morning
SAR1A	A_23_P127175	0.8761	0.131 ± 0.031	13.58	Morning
CENPN	A_23_P88740	0.8758	0.228 ± 0.081	-0.24	Evening
SOLH	A_24_P928639	0.8757	0.381 ± 0.174	-2.28	Evening
TPM1	A_32_P89709	0.8753	0.242 ± 0.075	17.87	Evening
GPR37	A_23_P145995	0.8752	0.615 ± 0.155	-2.54	Evening
POU6F2	A_23_P147900	0.8751	0.531 ± 0.151	2.48	Evening
LOC162632	A_32_P58796	0.8748	0.347 ± 0.116	8.69	Morning
A_32_P112531	A_32_P112531	0.8747	0.216 ± 0.073	-1.81	Evening
PPFIBP1	A_23_P337917	0.8746	0.354 ± 0.045	10.46	Morning
AK025669	A_32_P20912	0.8746	0.269 ± 0.073	8.07	Morning
COPG2	A_23_P61280	0.8743	0.239 ± 0.074	0.10	Evening
LAIR1	A_24_P262688	0.8740	0.388 ± 0.087	-2.92	Evening
SKA2	A_23_P301079	0.8740	0.143 ± 0.037	17.96	Evening
UCP2	A_23_P47704	0.8732	0.276 ± 0.076	-4.08	Evening

A_32_P212373	A_32_P212373	0.8732	0.235 ± 0.055	-0.31	Evening
PER3	CPID_442/A_24_P291231	0.8731	1.326 ± 0.117	7.23	Morning
E01827	A_24_P290314	0.8730	0.309 ± 0.124	0.33	Evening
B9D1	A_23_P152678	0.8730	0.245 ± 0.067	-2.42	Evening
UTP14A	A_24_P170538	0.8729	0.083 ± 0.04	15.23	Evening
COTL1	A_24_P416131	0.8718	0.13 ± 0.057	-4.68	Evening
TMEM107	A_24_P284353	0.8715	0.076 ± 0.028	12.29	Morning
PCNT	A_23_P57347	0.8713	0.09 ± 0.027	2.84	Morning
CLK3	A_32_P20691	0.8712	0.124 ± 0.059	-4.15	Evening
A_24_P771278	A_24_P771278	0.8711	0.463 ± 0.094	-2.34	Evening
A_24_P272515	A_24_P272515	0.8709	0.349 ± 0.099	1.85	Evening
ZRANB2	A_23_P85521	0.8708	0.207 ± 0.041	8.82	Morning
A_24_P384196	A_24_P384196	0.8707	0.394 ± 0.089	-0.78	Evening
CIDEA	A_23_P376704	0.8703	0.211 ± 0.093	-2.88	Evening
FAM86B2	A_24_P280868	0.8701	0.168 ± 0.043	0.12	Evening
A_24_P401582	A_24_P401582	0.8699	0.18 ± 0.059	-2.84	Evening
ATXN10	A_24_P391368	0.8696	0.258 ± 0.054	-3.16	Evening
BC035091	A_24_P576219	0.8696	0.415 ± 0.087	7.63	Morning
NR1D2	A_23_P302709	0.8695	0.707 ± 0.054	7.74	Morning
MYO7A	A_23_P422350	0.8694	0.39 ± 0.088	-3.43	Evening
SHPK	A_23_P310350	0.8693	0.116 ± 0.028	7.11	Morning
CENPQ	A_23_P70328	0.8692	0.114 ± 0.051	-3.14	Evening
DLAT	A_24_P372672	0.8691	0.159 ± 0.064	-4.86	Evening
WDR6	A_23_P95553	0.8690	0.218 ± 0.062	7.98	Morning
PER3	CPID_439/A_24_P291231	0.8690	1.429 ± 0.126	7.83	Morning
SMURF2	A_23_P100754	0.8687	0.167 ± 0.061	-4.99	Evening
MRPS10	A_23_P70417	0.8686	0.288 ± 0.05	-2.70	Evening
PDPK1	A_23_P66219	0.8685	0.087 ± 0.04	5.44	Morning
SUCLA2	A_24_P181275	0.8681	0.276 ± 0.081	-1.18	Evening
GCLC	A_23_P352879	0.8679	0.237 ± 0.09	-0.17	Evening
CNTN2	A_24_P114142	0.8674	0.467 ± 0.144	-2.14	Evening
DHRS1	A_24_P133475	0.8672	0.074 ± 0.036	3.80	Morning
GIN1	A_23_P110611	0.8672	0.104 ± 0.026	-3.49	Evening
Z25424	A_24_P938135	0.8669	0.214 ± 0.066	-1.87	Evening
SNX31	A_23_P382811	0.8661	0.365 ± 0.161	11.20	Morning
LOC643997	A_24_P366415	0.8660	0.164 ± 0.079	-1.13	Evening
A_32_P225209	A_32_P225209	0.8659	0.35 ± 0.091	9.45	Morning
FLJ33360	A_24_P307724	0.8658	0.377 ± 0.168	9.89	Morning
KDELR2	A_23_P19938	0.8657	0.08 ± 0.035	-3.99	Evening
ENST00000440728	A_24_P203658	0.8657	0.171 ± 0.083	0.15	Evening
A_32_P226700	A_32_P226700	0.8657	0.18 ± 0.046	-2.61	Evening
ALDH9A1	A_24_P385280	0.8657	0.126 ± 0.052	2.98	Morning
ENST00000331146	A_24_P229066	0.8655	0.247 ± 0.093	-1.21	Evening
A_24_P229616	A_24_P229616	0.8651	0.177 ± 0.061	-2.45	Evening
SH3BGR	A_23_P91520	0.8650	0.185 ± 0.069	-2.47	Evening
CDRT4	A_23_P427083	0.8648	0.173 ± 0.059	-0.01	Evening

CR602210	A_24_P923102	0.8647	0.226 ± 0.101	17.84	Evening
MKKS	A_24_P100664	0.8645	0.092 ± 0.043	-1.63	Evening
AA666384	A_32_P23731	0.8643	0.616 ± 0.161	8.51	Morning
GIMAP8	A_23_P168388	0.8642	0.145 ± 0.053	10.26	Morning
ZNF658	A_23_P419202	0.8639	0.138 ± 0.031	9.12	Morning
LOC100286918	A_24_P49421	0.8638	0.335 ± 0.101	-0.81	Evening
LOC388242	A_32_P135336	0.8638	0.17 ± 0.07	7.37	Morning
ASB8	A_23_P22200	0.8637	0.055 ± 0.024	-3.43	Evening
MYCBPAP	A_23_P141415	0.8636	0.149 ± 0.067	4.93	Morning
YTHDC2	A_23_P41732	0.8634	0.136 ± 0.046	10.94	Morning
ANKRD20A2	A_32_P66222	0.8633	0.223 ± 0.077	9.01	Morning
PER3	CPID_441/A_24_P291231	0.8630	1.273 ± 0.113	7.43	Morning
BI013473	A_32_P122285	0.8630	0.158 ± 0.049	1.40	Evening
GLYAT	A_23_P403886	0.8630	0.172 ± 0.084	-0.57	Evening
MCCC2	A_23_P18887	0.8630	0.089 ± 0.039	-3.05	Evening
BCAP31	A_24_P134683	0.8629	0.128 ± 0.041	-2.36	Evening
HSD11B1	A_23_P63209	0.8628	0.191 ± 0.085	-1.34	Evening
PKIA	A_23_P31765	0.8623	0.224 ± 0.069	-0.89	Evening
TNFRSF21	A_23_P30666	0.8622	0.236 ± 0.065	15.56	Evening
DYNLL1	A_23_P65031	0.8622	0.117 ± 0.04	-2.39	Evening
SRXN1	A_23_P320113	0.8621	0.092 ± 0.041	14.94	Evening
ALDH1L1	A_23_P258887	0.8621	0.195 ± 0.072	-3.13	Evening
A_24_P127063	A_24_P127063	0.8619	0.121 ± 0.049	-2.24	Evening
A_24_P306527	A_24_P306527	0.8617	0.308 ± 0.114	-0.17	Evening
TFDP1	A_32_P199301	0.8614	0.245 ± 0.04	-4.73	Evening
TNFRSF10C	A_23_P256724	0.8609	0.169 ± 0.084	-3.91	Evening
AK023328	A_24_P462725	0.8609	0.393 ± 0.08	8.56	Morning
A_24_P418106	A_24_P418106	0.8609	0.202 ± 0.073	-0.99	Evening
PTN	A_24_P870620	0.8607	0.401 ± 0.117	5.46	Morning
FLJ43681	A_32_P29582	0.8606	0.12 ± 0.02	-1.18	Evening
NPAS2	CPID_100/A_23_P218597	0.8605	1.176 ± 0.121	0.84	Evening
UBP1	A_23_P211738	0.8602	0.099 ± 0.027	13.81	Evening
A_24_P408981	A_24_P408981	0.8599	0.349 ± 0.081	-1.90	Evening
HNRNPA3	A_24_P706312	0.8599	0.11 ± 0.04	7.67	Morning
A_24_P375405	A_24_P375405	0.8597	0.175 ± 0.083	1.70	Evening
TBC1D20	A_23_P354187	0.8592	0.152 ± 0.034	-4.36	Evening
DNAJB12	A_23_P52382	0.8590	0.077 ± 0.038	17.28	Evening
CHPF2	A_32_P54442	0.8589	0.144 ± 0.032	10.17	Morning
DYNLL2	A_23_P54991	0.8587	0.112 ± 0.041	-3.23	Evening
LOC401127	A_23_P41267	0.8586	0.196 ± 0.044	-2.04	Evening
AL162073	A_32_P128391	0.8585	0.123 ± 0.061	-0.26	Evening
A_24_P789842	A_24_P789842	0.8583	0.158 ± 0.076	1.87	Evening
TMEM129	A_23_P354175	0.8580	0.21 ± 0.057	-4.10	Evening
PER3	CPID_471/A_24_P291231	0.8579	0.944 ± 0.088	10.11	Morning
TMEM52	A_23_P429624	0.8578	0.265 ± 0.1	-1.85	Evening
ALDH4A1	A_24_P263036	0.8577	0.133 ± 0.045	-5.17	Evening

DQ786246	A_24_P3704	0.8574	0.158 ± 0.047	7.30	Morning
CRY2	CPID_282/A_23_P388027	0.8572	0.349 ± 0.039	9.71	Morning
BC035371	A_24_P376422	0.8572	0.235 ± 0.09	9.79	Morning
DHDDS	A_24_P76805	0.8570	0.153 ± 0.057	-2.29	Evening
NUDT18	A_23_P123454	0.8568	0.151 ± 0.055	-3.73	Evening
NPAS2	CPID_104/A_23_P218597	0.8568	1.09 ± 0.116	0.92	Evening
ME1	A_23_P8196	0.8567	0.162 ± 0.066	-3.11	Evening
AK125162	A_32_P175935	0.8567	0.305 ± 0.117	-1.03	Evening
SERP1	A_24_P405552	0.8564	0.291 ± 0.044	-4.41	Evening
CRY2	CPID_287/A_23_P388027	0.8564	0.319 ± 0.038	9.43	Morning
ZBTB1	A_23_P99693	0.8561	0.187 ± 0.084	9.11	Morning
CRY2	CPID_284/A_23_P388027	0.8561	0.349 ± 0.041	9.72	Morning
PDE4DIP	A_23_P149153	0.8560	0.292 ± 0.069	-1.83	Evening
MUC20	A_23_P92225	0.8559	0.3 ± 0.109	9.52	Morning
SLC2A4	A_32_P151263	0.8559	0.129 ± 0.064	-5.91	Evening
C21orf81	A_32_P68942	0.8557	0.429 ± 0.151	10.04	Morning
PER3	CPID_438/A_24_P291231	0.8556	1.541 ± 0.145	7.16	Morning
NPAS2	CPID_97/A_23_P218597	0.8551	1.093 ± 0.119	0.76	Evening
FAM98A	A_23_P313728	0.8549	0.229 ± 0.044	-3.04	Evening
MTERFD2	A_24_P817066	0.8549	0.103 ± 0.037	7.89	Morning
JRK	A_23_P334635	0.8547	0.298 ± 0.147	-3.26	Evening
ABCA3	A_23_P140876	0.8546	0.115 ± 0.036	-1.94	Evening
C6orf141	A_32_P114483	0.8544	0.185 ± 0.062	8.89	Morning
ZNF259P	A_24_P213206	0.8544	0.45 ± 0.113	-0.76	Evening
ZBED5	A_24_P920319	0.8543	0.19 ± 0.082	0.08	Evening
PER3	CPID_473/A_24_P291231	0.8542	0.934 ± 0.089	10.22	Morning
CDON	A_32_P172141	0.8541	0.2 ± 0.056	1.46	Evening
EIF2C1	A_23_P11764	0.8541	0.035 ± 0.018	-3.68	Evening
KHDC1	A_24_P20814	0.8540	0.436 ± 0.103	-0.89	Evening
LOC100270746	A_32_P232035	0.8540	0.232 ± 0.042	9.46	Morning
A_24_P75308	A_24_P75308	0.8539	0.128 ± 0.063	-2.36	Evening
KCTD10	A_23_P162540	0.8538	0.088 ± 0.04	16.61	Evening
C21orf122	A_23_P143514	0.8537	0.227 ± 0.062	-2.48	Evening
A_24_P840868	A_24_P840868	0.8537	0.232 ± 0.062	-1.96	Evening
ALDOAP2	A_24_P740942	0.8536	0.319 ± 0.06	-2.39	Evening
MPV17	A_23_P143089	0.8533	0.168 ± 0.048	-1.99	Evening
ARNTL	A_23_P162037	0.8533	1.247 ± 0.112	-1.02	Evening
A_32_P177843	A_32_P177843	0.8531	0.53 ± 0.114	8.37	Morning
A_23_P64962	A_23_P64962	0.8531	0.266 ± 0.077	7.55	Morning
MAP2K6	A_23_P207445	0.8529	0.271 ± 0.062	15.81	Evening
PER3	CPID_472/A_24_P291231	0.8529	0.921 ± 0.087	10.17	Morning
OR2A9P	A_23_P123172	0.8529	0.256 ± 0.127	5.03	Morning
CRY2	CPID_286/A_23_P388027	0.8527	0.326 ± 0.038	9.42	Morning
A_24_P196019	A_24_P196019	0.8525	0.162 ± 0.049	-3.75	Evening
PGA3	A_23_P150547	0.8524	0.3 ± 0.079	-5.52	Evening
PER3	CPID_477/A_24_P291231	0.8522	0.955 ± 0.09	10.21	Morning

MME	A_24_P260101	0.8521	0.249 ± 0.099	-1.91	Evening
EIF2A	A_32_P197524	0.8518	0.262 ± 0.103	8.41	Morning
DAPK2	A_24_P10233	0.8518	0.174 ± 0.052	6.58	Morning
CRY2	CPID_281/A_23_P388027	0.8514	0.35 ± 0.04	9.58	Morning
FAH	A_23_P129221	0.8514	0.206 ± 0.071	-2.42	Evening
BC022928	A_24_P706371	0.8511	0.185 ± 0.072	-1.54	Evening
FGFBP2	A_24_P226069	0.8507	0.502 ± 0.128	-2.79	Evening
OSGIN2	A_24_P261083	0.8506	0.164 ± 0.078	-5.25	Evening
ARNTL	CPID_534/A_23_P162037	0.8505	1.2 ± 0.11	-1.00	Evening
HLF	A_23_P356585	0.8502	0.491 ± 0.058	10.25	Morning
PPP1R16A	A_24_P9605	0.8498	0.163 ± 0.073	-1.21	Evening
PLCE1	A_23_P35617	0.8498	0.203 ± 0.08	-4.13	Evening
ARL17P1	A_23_P371885	0.8496	0.431 ± 0.111	9.76	Morning
C20orf12	A_24_P408297	0.8490	0.555 ± 0.108	0.56	Evening
CK300181	A_32_P19840	0.8489	0.201 ± 0.081	-2.52	Evening
PDE11A	A_32_P116857	0.8488	0.192 ± 0.08	3.63	Morning
CTNND1	A_23_P251316	0.8488	0.134 ± 0.032	4.78	Morning
PER2	A_23_P411162	0.8486	0.829 ± 0.091	10.53	Morning
KIAA0040	A_24_P79617	0.8484	0.3 ± 0.112	17.76	Evening
A_23_P89841	A_23_P89841	0.8484	0.332 ± 0.095	8.47	Morning
PER3	CPID_476/A_24_P291231	0.8484	0.959 ± 0.093	10.25	Morning
PER3	CPID_451/A_24_P291231	0.8483	1.386 ± 0.138	7.19	Morning
TRIT1	A_23_P12062	0.8483	0.128 ± 0.038	8.33	Morning
CABLES1	A_23_P422851	0.8482	0.436 ± 0.061	2.87	Morning
PER3	CPID_440/A_24_P291231	0.8480	1.207 ± 0.112	7.64	Morning
HSPB8	A_23_P162579	0.8478	0.241 ± 0.06	-4.53	Evening
TSLP	A_23_P121987	0.8477	0.279 ± 0.084	-2.34	Evening
PBX1	A_32_P25397	0.8476	0.176 ± 0.053	-3.42	Evening
LRRC37B2	A_24_P144983	0.8471	0.126 ± 0.044	8.79	Morning
A_24_P213144	A_24_P213144	0.8470	0.413 ± 0.084	-2.80	Evening
FAAH	A_23_P103226	0.8469	0.191 ± 0.05	-5.75	Evening
A_24_P307126	A_24_P307126	0.8468	0.477 ± 0.103	-3.51	Evening
PEX1	A_23_P20045	0.8466	0.109 ± 0.019	6.69	Morning
A_24_P931282	A_24_P931282	0.8465	0.305 ± 0.091	-2.01	Evening
A_24_P853302	A_24_P853302	0.8465	0.441 ± 0.121	-1.32	Evening
A_24_P307205	A_24_P307205	0.8460	0.318 ± 0.112	0.80	Evening
BET1L	A_23_P127879	0.8458	0.149 ± 0.051	-3.49	Evening
BX111592	A_32_P49959	0.8458	0.216 ± 0.109	9.77	Morning
MRPL48	A_23_P162106	0.8455	0.139 ± 0.045	-1.21	Evening
RPL23AP7	A_23_P256051	0.8455	0.211 ± 0.066	-1.85	Evening
ARNTL	CPID_531/A_23_P162037	0.8454	1.281 ± 0.118	-0.95	Evening
A_24_P692030	A_24_P692030	0.8453	0.292 ± 0.081	-1.82	Evening
NUDT13	A_32_P41471	0.8453	0.164 ± 0.08	5.27	Morning
EDC3	A_23_P21155	0.8449	0.171 ± 0.037	-4.64	Evening
STK17A	A_23_P82550	0.8447	0.174 ± 0.07	14.91	Evening
PER3	CPID_474/A_24_P291231	0.8446	0.943 ± 0.092	10.10	Morning

FGF13	A_23_P217319	0.8445	0.136 ± 0.053	-5.38	Evening
CS	A_23_P47818	0.8445	0.103 ± 0.049	-3.03	Evening
TPM3	A_24_P58579	0.8444	0.073 ± 0.028	-2.83	Evening
AK129982	A_32_P145080	0.8443	0.345 ± 0.061	7.78	Morning
A_23_P149270	A_23_P149270	0.8441	0.316 ± 0.061	-3.84	Evening
C2orf89	A_23_P56703	0.8441	0.2 ± 0.087	-0.26	Evening
CDC2L2	A_24_P937644	0.8441	0.214 ± 0.088	8.68	Morning
TXNL4A	A_23_P130304	0.8439	0.113 ± 0.03	-3.44	Evening
SMUG1	A_24_P75072	0.8437	0.108 ± 0.053	-2.66	Evening
KDSR	A_32_P515088	0.8437	0.168 ± 0.047	-3.93	Evening
SURF4	A_23_P32052	0.8435	0.119 ± 0.032	-3.98	Evening
PHF13	A_32_P58425	0.8433	0.217 ± 0.038	5.01	Morning
ACOT1	A_24_P161036	0.8431	0.134 ± 0.066	-2.48	Evening
NCDN	A_23_P97736	0.8428	0.165 ± 0.047	-2.82	Evening
PER3	A_24_P230948	0.8427	0.965 ± 0.089	10.02	Morning
FTHL17	A_23_P148410	0.8427	0.369 ± 0.099	-3.32	Evening
AKR7A3	A_23_P103968	0.8425	0.121 ± 0.055	-3.29	Evening
GSTM5	A_24_P212021	0.8424	0.391 ± 0.157	-2.83	Evening
IMPAD1	A_23_P136232	0.8423	0.194 ± 0.054	-3.57	Evening
LGALS12	A_23_P139198	0.8423	0.154 ± 0.078	2.67	Evening
PEX13	A_24_P11587	0.8422	0.107 ± 0.049	-2.39	Evening
MXRA7	A_32_P161681	0.8422	0.127 ± 0.062	-3.70	Evening
SNRPB2	A_24_P194260	0.8418	0.137 ± 0.056	-3.49	Evening
ATP6V1G2	A_23_P500410	0.8418	0.167 ± 0.081	-3.36	Evening
LOC644563	A_24_P127121	0.8418	0.116 ± 0.04	-2.58	Evening
NARS2	A_23_P24960	0.8417	0.15 ± 0.049	-1.00	Evening
TRUB2	A_23_P169428	0.8417	0.089 ± 0.039	0.76	Evening
PSCA	A_23_P71379	0.8416	0.213 ± 0.093	5.42	Morning
CRY2	CPID_283/A_23_P388027	0.8415	0.327 ± 0.042	9.64	Morning
CCDC122	A_32_P213661	0.8415	0.132 ± 0.067	6.34	Morning
CRY2	CPID_290/A_23_P388027	0.8412	0.327 ± 0.038	9.43	Morning
LOC400061	A_24_P92973	0.8406	0.222 ± 0.09	0.21	Evening
A_24_P229911	A_24_P229911	0.8404	0.089 ± 0.028	-4.73	Evening
LOC100288755	A_32_P172917	0.8404	0.091 ± 0.044	4.81	Morning
REV1	A_24_P291426	0.8404	0.097 ± 0.027	10.62	Morning
PPIL5	A_24_P13533	0.8403	0.173 ± 0.054	-5.77	Evening
FNTB	A_24_P124831	0.8403	0.244 ± 0.055	-3.16	Evening
SRA1	A_23_P257578	0.8402	0.078 ± 0.031	-4.95	Evening
BC001335	A_24_P145107	0.8401	0.155 ± 0.051	8.70	Morning
PER3	CPID_475/A_24_P291231	0.8400	0.935 ± 0.093	10.23	Morning
TK2	A_24_P219324	0.8398	0.236 ± 0.05	-4.16	Evening
A_24_P920388	A_24_P920388	0.8396	0.246 ± 0.103	-1.48	Evening
CSTA	A_23_P41114	0.8396	0.253 ± 0.071	-1.07	Evening
ACTR3C	A_24_P76635	0.8396	0.146 ± 0.067	-1.85	Evening
SUB1	A_24_P320328	0.8395	0.088 ± 0.044	16.15	Evening
A_24_P736638	A_24_P736638	0.8394	0.231 ± 0.063	-2.37	Evening

SRA1	A_24_P274607	0.8394	0.107 ± 0.036	-2.77	Evening
MRPL53	A_24_P371194	0.8391	0.099 ± 0.048	-2.29	Evening
GLYAT	A_23_P75749	0.8389	0.398 ± 0.084	1.29	Evening
ACADS	A_23_P65022	0.8388	0.346 ± 0.066	-2.85	Evening
FAM173B	A_23_P133279	0.8387	0.107 ± 0.048	-2.21	Evening
NPAS2	CPID_98/A_23_P218597	0.8386	1.143 ± 0.131	0.86	Evening
RNF135	A_23_P252283	0.8384	0.07 ± 0.031	2.75	Evening
MICB	A_23_P387471	0.8384	0.154 ± 0.061	-4.86	Evening
A_24_P170203	A_24_P170203	0.8383	0.083 ± 0.042	0.14	Evening
A_24_P25020	A_24_P25020	0.8383	0.254 ± 0.094	-0.63	Evening
TMEM136	A_23_P329890	0.8383	0.163 ± 0.03	-3.07	Evening
SENP6	A_24_P106382	0.8381	0.189 ± 0.039	5.72	Morning
BC029473	A_32_P123293	0.8379	0.347 ± 0.153	9.27	Morning
BG187898	A_24_P204515	0.8379	0.12 ± 0.047	-1.58	Evening
ARNTL	CPID_529/A_23_P162037	0.8377	1.231 ± 0.117	-0.85	Evening
A_32_P106864	A_32_P106864	0.8373	0.136 ± 0.052	3.51	Morning
ALMS1P	A_23_P435657	0.8371	0.183 ± 0.074	-1.65	Evening
RPL24	A_32_P100974	0.8369	0.327 ± 0.135	-0.77	Evening
RPL22	A_24_P938169	0.8367	0.339 ± 0.084	-1.81	Evening
BU595528	A_32_P174214	0.8363	0.136 ± 0.048	-3.49	Evening
LOC344328	A_24_P24912	0.8361	0.232 ± 0.103	1.27	Evening
GOLGA8E	A_32_P103669	0.8360	0.209 ± 0.107	12.04	Morning
HIST2H2BE	A_24_P148321	0.8358	0.364 ± 0.08	-2.78	Evening
ECH1	A_23_P153853	0.8356	0.241 ± 0.075	-1.43	Evening
IMPAD1	A_24_P240732	0.8356	0.211 ± 0.049	-5.07	Evening
ENST00000450815	A_23_P109677	0.8356	0.17 ± 0.054	-2.90	Evening
RAB7B	A_23_P61590	0.8354	0.54 ± 0.09	0.09	Evening
CR617560	A_32_P85279	0.8353	0.085 ± 0.031	0.85	Evening
A_24_P84112	A_24_P84112	0.8353	0.173 ± 0.044	-3.16	Evening
LOC100133161	A_24_P818010	0.8351	0.199 ± 0.096	7.49	Morning
PIGC	A_23_P74852	0.8351	0.254 ± 0.07	-5.74	Evening
NPAS2	A_23_P218597	0.8350	0.696 ± 0.103	1.65	Evening
BLVRB	A_23_P153351	0.8350	0.139 ± 0.062	-2.65	Evening
A_24_P126902	A_24_P126902	0.8350	0.269 ± 0.092	-0.13	Evening
CYP2A7	A_23_P27528	0.8349	0.378 ± 0.111	-2.47	Evening
MAP6	A_23_P47728	0.8349	0.188 ± 0.073	-0.20	Evening
PER1	CPID_592/A_23_P89589	0.8349	1.282 ± 0.135	11.05	Morning
CHST4	A_23_P141035	0.8348	0.341 ± 0.148	11.93	Morning
GPRASP1	A_23_P96590	0.8347	0.14 ± 0.053	11.25	Morning
SNRNP27	A_23_P39774	0.8347	0.092 ± 0.04	-4.99	Evening
CCDC94	A_23_P130865	0.8346	0.095 ± 0.043	-0.55	Evening
DHDDS	A_24_P179769	0.8345	0.121 ± 0.048	-3.04	Evening
UBE2L3	A_23_P166353	0.8345	0.121 ± 0.026	-4.26	Evening
TSPAN33	A_24_P365901	0.8339	0.275 ± 0.071	-3.65	Evening
CDC2L2	A_24_P96709	0.8338	0.157 ± 0.04	-2.54	Evening
A_24_P598919	A_24_P598919	0.8338	0.197 ± 0.062	-5.19	Evening

FMO2	A_23_P355295	0.8337	0.256 ± 0.094	13.84	Evening
A_24_P384210	A_24_P384210	0.8335	0.478 ± 0.147	-0.12	Evening
NPAS2	CPID_96/A_23_P218597	0.8334	1.255 ± 0.136	1.17	Evening
A_24_P67432	A_24_P67432	0.8332	0.176 ± 0.058	-1.91	Evening
SCN3B	A_24_P312790	0.8330	0.377 ± 0.191	-0.11	Evening
A_23_P89680	A_23_P89680	0.8329	0.494 ± 0.123	9.09	Morning
AK092810	A_24_P696507	0.8328	0.098 ± 0.036	1.58	Evening
MPI	A_23_P60579	0.8326	0.137 ± 0.041	-1.02	Evening
DYNLL1	A_24_P124672	0.8326	0.111 ± 0.048	-1.07	Evening
BC013295	A_24_P170874	0.8323	0.228 ± 0.081	13.32	Morning
SCAPER	A_24_P162244	0.8323	0.261 ± 0.104	7.85	Morning
A_23_P203702	A_23_P203702	0.8322	0.1 ± 0.045	12.94	Morning
C20orf12	A_23_P40315	0.8320	0.456 ± 0.115	0.72	Evening
SCARB1	A_23_P203900	0.8318	0.188 ± 0.051	11.17	Morning
ATXN3	A_23_P129031	0.8318	0.178 ± 0.04	-1.95	Evening
WDR3	A_23_P23318	0.8317	0.112 ± 0.052	-2.08	Evening
PBLD	A_24_P312519	0.8315	0.195 ± 0.056	-5.91	Evening
KANK4	A_32_P51237	0.8314	0.271 ± 0.118	12.56	Morning
A_24_P821146	A_24_P821146	0.8313	0.289 ± 0.092	0.92	Evening
BMS1	A_24_P649747	0.8312	0.137 ± 0.064	12.93	Morning
LHPP	A_23_P75299	0.8311	0.109 ± 0.043	0.77	Evening
PHF8	A_24_P942253	0.8307	0.139 ± 0.031	7.39	Morning
PDE11A	A_32_P228037	0.8306	0.24 ± 0.094	1.13	Evening
DNLZ	A_23_P216836	0.8303	0.078 ± 0.04	-1.02	Evening
NPAS2	CPID_95/A_23_P218597	0.8302	1.057 ± 0.125	0.94	Evening
A_24_P501698	A_24_P501698	0.8301	0.24 ± 0.115	0.12	Evening
GGNBP2	A_24_P67892	0.8300	0.084 ± 0.024	2.80	Evening
COL10A1	A_23_P214144	0.8299	0.43 ± 0.112	9.59	Morning
A_24_P307486	A_24_P307486	0.8298	0.165 ± 0.055	-3.17	Evening
GYG2	A_32_P111701	0.8298	0.224 ± 0.084	-3.92	Evening
A_24_P32715	A_24_P32715	0.8296	0.283 ± 0.117	-4.04	Evening
AK057088	A_24_P554156	0.8293	0.126 ± 0.037	8.45	Morning
A_24_P126931	A_24_P126931	0.8290	0.304 ± 0.097	-0.97	Evening
ALDOA	A_23_P88963	0.8290	0.125 ± 0.044	-2.47	Evening
LOC149134	A_24_P803885	0.8289	0.281 ± 0.092	8.79	Morning
A_24_P238427	A_24_P238427	0.8288	0.359 ± 0.12	0.92	Evening
KIF5B	A_23_P86403	0.8285	0.112 ± 0.053	16.39	Evening
MICB	A_32_P74983	0.8283	0.214 ± 0.067	10.03	Morning
SPG21	A_23_P147450	0.8283	0.133 ± 0.047	-2.13	Evening
SDSL	A_23_P53439	0.8283	0.209 ± 0.095	-0.70	Evening
PRELP	A_23_P51499	0.8280	0.236 ± 0.081	-0.36	Evening
BMS1	A_32_P11450	0.8278	0.163 ± 0.082	11.28	Morning
CRY2	CPID_289/A_23_P388027	0.8276	0.306 ± 0.039	9.47	Morning
SHMT1	A_24_P205364	0.8276	0.181 ± 0.064	-4.63	Evening
ZNF629	A_23_P163816	0.8275	0.142 ± 0.031	9.20	Morning
CRY2	CPID_288/A_23_P388027	0.8275	0.32 ± 0.041	9.53	Morning

BOLA3	A_23_P131706	0.8273	0.138 ± 0.052	-1.68	Evening
TRAF7	A_23_P206474	0.8271	0.325 ± 0.068	-3.30	Evening
MRPL10	A_23_P218423	0.8271	0.11 ± 0.033	-2.82	Evening
A_24_P75994	A_24_P75994	0.8269	0.356 ± 0.142	10.48	Morning
A_23_P5171	A_23_P5171	0.8268	0.082 ± 0.041	-1.80	Evening
PTPN21	A_23_P77031	0.8268	0.089 ± 0.045	-5.31	Evening
EIF4E2	A_24_P934755	0.8268	0.273 ± 0.045	-4.60	Evening
A_24_P606663	A_24_P606663	0.8266	0.218 ± 0.109	0.79	Evening
SLC14A2	A_24_P136471	0.8264	0.278 ± 0.121	-2.22	Evening
ARNTL	CPID_533/A_23_P162037	0.8262	1.219 ± 0.121	-0.90	Evening
DHCR24	A_23_P379475	0.8262	0.285 ± 0.139	0.49	Evening
CDH23	A_23_P217946	0.8261	0.345 ± 0.099	-3.07	Evening
A_32_P171225	A_32_P171225	0.8260	0.261 ± 0.098	-0.70	Evening
A_24_P745670	A_24_P745670	0.8260	0.223 ± 0.069	-2.02	Evening
ADAMTS2	A_23_P213615	0.8258	0.159 ± 0.067	-3.29	Evening
SLC9A6	A_23_P22625	0.8257	0.109 ± 0.023	-3.41	Evening
ENST00000275524	A_32_P232543	0.8252	0.196 ± 0.082	1.26	Evening
UBTD1	A_23_P161501	0.8252	0.185 ± 0.079	-2.76	Evening
TALDO1	A_32_P18258	0.8251	0.152 ± 0.036	-3.00	Evening
DGAT2	A_24_P295791	0.8251	0.309 ± 0.108	15.63	Evening
PER1	CPID_590/A_23_P89589	0.8250	1.22 ± 0.136	11.09	Morning
AK056365	A_24_P926554	0.8249	0.264 ± 0.117	17.39	Evening
ALDH4A1	A_32_P192970	0.8249	0.502 ± 0.093	-4.00	Evening
ARNTL	CPID_530/A_23_P162037	0.8248	1.284 ± 0.122	-0.68	Evening
AMACR	A_24_P106297	0.8247	0.21 ± 0.095	-0.68	Evening
A_24_P186779	A_24_P186779	0.8244	0.347 ± 0.145	-0.85	Evening
BC001335	A_24_P145103	0.8244	0.117 ± 0.041	7.54	Morning
PER3	CPID_468/A_24_P291231	0.8242	0.894 ± 0.092	10.37	Morning
RNF141	A_23_P139066	0.8242	0.135 ± 0.039	-3.65	Evening
MDH2	A_24_P46808	0.8241	0.119 ± 0.039	-0.86	Evening
AP3S2	A_24_P287691	0.8240	0.132 ± 0.039	-4.67	Evening
ESAM	A_24_P13190	0.8240	0.275 ± 0.093	-4.66	Evening
CRY2	A_23_P127394	0.8239	0.3 ± 0.04	9.76	Morning
SLC1A4	A_23_P91151	0.8237	0.182 ± 0.068	17.29	Evening
PER1	CPID_587/A_23_P89589	0.8236	1.219 ± 0.137	11.07	Morning
KDELR2	A_24_P42517	0.8236	0.117 ± 0.042	-4.08	Evening
PER3	CPID_449/A_24_P291231	0.8236	1.144 ± 0.131	7.24	Morning
ACOT2	A_24_P301195	0.8235	0.207 ± 0.082	-2.47	Evening
MXRA7	A_24_P810697	0.8230	0.244 ± 0.089	-3.10	Evening
NCLN	A_23_P130900	0.8229	0.181 ± 0.062	-2.45	Evening
CAMKK1	A_23_P431933	0.8228	0.418 ± 0.096	8.62	Morning
DBNDD1	A_23_P26439	0.8227	0.412 ± 0.137	-5.38	Evening
BC019599	A_32_P218251	0.8227	0.201 ± 0.08	4.37	Morning
ACADSB	A_23_P158570	0.8226	0.207 ± 0.052	-3.83	Evening
ADAL	A_32_P221694	0.8226	0.198 ± 0.051	9.33	Morning
PER1	A_23_P89589	0.8225	1.21 ± 0.134	11.09	Morning

STAC	A_24_P234415	0.8225	0.389 ± 0.133	-0.12	Evening
C3orf31	A_23_P212160	0.8224	0.078 ± 0.027	3.88	Morning
PER3	CPID_450/A_24_P291231	0.8222	1.43 ± 0.138	7.60	Morning
C14orf143	A_23_P129014	0.8222	0.147 ± 0.069	-2.21	Evening
ARNTL	CPID_532/A_23_P162037	0.8222	1.257 ± 0.126	-0.95	Evening
PER2	CPID_1/A_23_P209320	0.8222	0.85 ± 0.099	11.06	Morning
NUDT1	A_23_P134295	0.8220	0.095 ± 0.039	-0.77	Evening
LOC100049716	A_23_P431569	0.8220	0.16 ± 0.06	-2.55	Evening
A_24_P255314	A_24_P255314	0.8219	0.28 ± 0.108	-0.42	Evening
RPP14	A_24_P839254	0.8219	0.082 ± 0.029	9.13	Morning
PER1	CPID_584/A_23_P89589	0.8217	1.269 ± 0.138	11.15	Morning
C10orf78	A_23_P413193	0.8214	0.108 ± 0.032	-2.52	Evening
MMAB	A_24_P364381	0.8214	0.244 ± 0.049	-2.13	Evening
PER1	CPID_583/A_23_P89589	0.8214	1.221 ± 0.136	11.11	Morning
CDH23	A_23_P307968	0.8212	0.223 ± 0.096	17.11	Evening
A_32_P224926	A_32_P224926	0.8211	0.142 ± 0.069	8.34	Morning
BU191598	A_32_P199506	0.8210	0.169 ± 0.076	15.39	Evening
PPIL5	A_23_P88362	0.8209	0.155 ± 0.064	-2.27	Evening
STK10	A_24_P46417	0.8209	0.125 ± 0.058	-2.53	Evening
HNRNPR	A_23_P45726	0.8208	0.072 ± 0.028	-1.09	Evening
ARF1	A_24_P190894	0.8208	0.084 ± 0.026	-5.57	Evening
RNASE4	A_23_P205531	0.8208	0.141 ± 0.065	-1.79	Evening
BU929651	A_32_P128974	0.8207	0.133 ± 0.03	9.52	Morning
GBAP	A_32_P512061	0.8207	0.194 ± 0.057	-2.93	Evening
SNHG10	A_32_P82475	0.8206	0.203 ± 0.072	1.19	Evening
KLF13	A_32_P197489	0.8205	0.35 ± 0.045	11.44	Morning
NDUFAF1	A_23_P26254	0.8205	0.083 ± 0.041	-3.74	Evening
DHDPSL	A_23_P364613	0.8205	0.201 ± 0.082	-1.11	Evening
A_24_P238525	A_24_P238525	0.8204	0.205 ± 0.069	8.10	Morning
RRP7A	A_23_P29303	0.8198	0.219 ± 0.064	-3.21	Evening
PSORS1C1	A_23_P133902	0.8198	0.43 ± 0.157	5.50	Morning
A_24_P324506	A_24_P324506	0.8194	0.317 ± 0.09	-2.49	Evening
SLC25A6	A_23_P33752	0.8192	0.085 ± 0.037	-1.97	Evening
A_24_P93452	A_24_P93452	0.8190	0.254 ± 0.063	-1.62	Evening
TALDO1	A_23_P44993	0.8190	0.169 ± 0.051	-2.47	Evening
KCNIP2	A_23_P202189	0.8189	0.196 ± 0.078	-4.07	Evening
A_24_P32646	A_24_P32646	0.8189	0.185 ± 0.066	-3.29	Evening
A_24_P272713	A_24_P272713	0.8189	0.15 ± 0.072	-0.85	Evening
A_24_P451992	A_24_P451992	0.8187	0.439 ± 0.11	-2.69	Evening
AQP7P2	A_24_P281009	0.8186	0.187 ± 0.071	-2.58	Evening
EIF5	A_24_P398810	0.8186	0.204 ± 0.052	9.90	Morning
A_24_P174063	A_24_P174063	0.8183	0.235 ± 0.056	-1.85	Evening
TMEM8B	A_24_P317827	0.8180	0.083 ± 0.042	-2.25	Evening
SLC6A8	A_24_P23400	0.8180	0.18 ± 0.044	-5.58	Evening
SMN1	A_23_P58466	0.8180	0.101 ± 0.037	8.16	Morning
SRP9	A_32_P165340	0.8179	0.322 ± 0.104	-2.32	Evening

MYL12B	A_24_P75230	0.8179	0.08 ± 0.033	-4.70	Evening
A_24_P41551	A_24_P41551	0.8179	0.216 ± 0.082	0.11	Evening
SLC4A3	A_23_P39647	0.8178	0.147 ± 0.071	3.32	Morning
STK17A	A_24_P337796	0.8176	0.17 ± 0.078	15.14	Evening
CCDC25	A_23_P71300	0.8176	0.133 ± 0.047	-1.88	Evening
GPX8	A_23_P122052	0.8175	0.151 ± 0.045	-4.02	Evening
C21orf57	A_32_P18547	0.8175	0.146 ± 0.062	-1.64	Evening
ALDH1B1	A_23_P135294	0.8174	0.146 ± 0.073	-2.84	Evening
LOC572558	A_32_P157208	0.8174	0.22 ± 0.086	2.83	Morning
PER1	CPID_585/A_23_P89589	0.8174	1.271 ± 0.14	11.10	Morning
ARNTL	CPID_528/A_23_P162037	0.8173	1.35 ± 0.136	-0.88	Evening
ARMC6	A_23_P425750	0.8173	0.111 ± 0.036	-1.73	Evening
RASL10B	A_24_P322229	0.8173	0.295 ± 0.1	-3.05	Evening
LOC644297	A_23_P164316	0.8173	0.392 ± 0.107	8.23	Morning
OMA1	A_23_P138139	0.8173	0.072 ± 0.033	0.56	Evening
C6orf108	A_24_P414733	0.8172	0.138 ± 0.054	-2.61	Evening
TFCP2	A_23_P347528	0.8171	0.184 ± 0.041	-0.64	Evening
LOC100190939	A_23_P326987	0.8170	0.258 ± 0.087	9.66	Morning
P2RY12	A_23_P143902	0.8167	0.194 ± 0.092	17.83	Evening
ERMAP	A_24_P105391	0.8166	0.17 ± 0.073	-3.17	Evening
A_32_P115451	A_32_P115451	0.8166	0.099 ± 0.032	6.99	Morning
STX6	A_23_P331992	0.8164	0.263 ± 0.045	6.03	Morning
H6PD	A_24_P529644	0.8164	0.238 ± 0.053	7.77	Morning
STK24	A_23_P60683	0.8163	0.141 ± 0.034	-4.74	Evening
MRO	A_23_P4545	0.8161	0.382 ± 0.106	-1.95	Evening
PDLIM5	A_24_P174641	0.8161	0.105 ± 0.048	-0.01	Evening
C22orf32	A_24_P271542	0.8161	0.12 ± 0.057	0.62	Evening
C6orf57	A_23_P256279	0.8160	0.084 ± 0.04	-2.71	Evening
RNLS	A_23_P202501	0.8160	0.284 ± 0.087	-0.24	Evening
LOC389831	A_24_P541576	0.8159	0.187 ± 0.085	12.34	Morning
ATPAF1	A_24_P229199	0.8157	0.188 ± 0.05	-4.02	Evening
AK023682	A_24_P166473	0.8156	0.243 ± 0.041	6.72	Morning
USP6	A_24_P170454	0.8154	0.291 ± 0.121	6.46	Morning
TPP1	A_24_P38815	0.8153	0.27 ± 0.04	-2.26	Evening
PER1	CPID_589/A_23_P89589	0.8153	1.222 ± 0.136	11.08	Morning
Mar-02	A_23_P33683	0.8152	0.208 ± 0.051	-3.48	Evening
OVOS2	A_23_P25069	0.8151	0.233 ± 0.073	5.03	Morning
P4HA2	A_23_P30363	0.8148	0.16 ± 0.036	-4.30	Evening
RP1-21O18.1	A_32_P139505	0.8147	0.369 ± 0.108	-2.37	Evening
A_24_P928017	A_24_P928017	0.8144	0.132 ± 0.054	-2.78	Evening
LOC644684	A_32_P169785	0.8143	0.129 ± 0.064	12.11	Morning
C13orf38	A_23_P14216	0.8143	0.273 ± 0.117	-1.64	Evening
ODC1	A_23_P165840	0.8143	0.28 ± 0.05	-3.89	Evening
A_32_P184304	A_32_P184304	0.8142	0.29 ± 0.101	13.34	Morning
FUNDC2	A_32_P18251	0.8141	0.323 ± 0.081	-1.93	Evening
EIF3L	A_23_P57521	0.8141	0.105 ± 0.037	-1.36	Evening

PER1	CPID_586/A_23_P89589	0.8140	1.243 ± 0.138	11.06	Morning
SLC24A6	A_23_P328600	0.8140	0.086 ± 0.043	13.23	Morning
UHRF2	A_23_P9144	0.8140	0.135 ± 0.033	-1.37	Evening
DNAJB4	A_24_P393958	0.8139	0.203 ± 0.049	17.98	Evening
CCDC144B	A_24_P75917	0.8138	0.287 ± 0.107	8.58	Morning
C11orf54	A_23_P202750	0.8137	0.086 ± 0.043	7.60	Morning
UGDH	A_23_P167067	0.8137	0.229 ± 0.055	-4.40	Evening
SMG1	A_24_P244952	0.8134	0.136 ± 0.042	0.62	Evening
PER1	CPID_591/A_23_P89589	0.8134	1.233 ± 0.14	11.09	Morning
ACSS1	A_23_P120594	0.8133	0.211 ± 0.052	10.05	Morning
A_24_P281683	A_24_P281683	0.8132	0.348 ± 0.11	-2.42	Evening
AK027091	A_23_P399292	0.8132	0.316 ± 0.138	9.77	Morning
FLJ45445	A_24_P898915	0.8131	0.247 ± 0.09	7.92	Morning
LOC407835	A_32_P150856	0.8128	0.046 ± 0.023	-3.59	Evening
LOC492303	A_32_P197620	0.8126	0.066 ± 0.031	-4.36	Evening
PCGF3	A_24_P388632	0.8126	0.123 ± 0.038	8.38	Morning
XPO6	A_23_P206822	0.8126	0.371 ± 0.147	1.45	Evening
BDP1	A_24_P940776	0.8124	0.088 ± 0.043	-3.50	Evening
CCDC48	A_23_P166566	0.8122	0.214 ± 0.062	10.84	Morning
CFLAR	A_23_P209394	0.8121	0.112 ± 0.042	14.97	Evening
BAK1	A_23_P145357	0.8121	0.081 ± 0.025	8.65	Morning
CYBASC3	A_23_P342131	0.8121	0.236 ± 0.073	-3.10	Evening
HNF1A	A_23_P36757	0.8120	0.179 ± 0.056	12.28	Morning
MLLT4	A_23_P436353	0.8119	0.246 ± 0.058	10.09	Morning
WARS2	A_23_P74380	0.8119	0.075 ± 0.038	1.41	Evening
HAAO	A_23_P28697	0.8118	0.271 ± 0.07	-2.92	Evening
BI597240	A_32_P108554	0.8118	0.135 ± 0.053	7.02	Morning
EHD1	A_24_P184295	0.8117	0.304 ± 0.049	-5.27	Evening
A_32_P190036	A_32_P190036	0.8114	0.128 ± 0.058	-0.17	Evening
CA3	A_23_P20316	0.8111	0.553 ± 0.245	1.94	Evening
A_32_P109078	A_32_P109078	0.8110	0.34 ± 0.127	9.36	Morning
ZNF826	A_24_P921554	0.8108	0.363 ± 0.184	0.75	Evening
RHOBTB3	A_23_P92710	0.8107	0.364 ± 0.075	0.20	Evening
PER1	CPID_588/A_23_P89589	0.8106	1.239 ± 0.143	11.11	Morning
ZDHHC14	A_23_P250619	0.8106	0.278 ± 0.057	7.93	Morning
AMIGO1	A_24_P302506	0.8105	0.179 ± 0.086	-1.47	Evening
IMMT	A_23_P39844	0.8104	0.059 ± 0.027	-3.41	Evening
TRIL	A_24_P183664	0.8102	0.283 ± 0.063	0.47	Evening
CPSF1	A_23_P169112	0.8100	0.094 ± 0.039	6.23	Morning
WDR45	A_23_P251717	0.8099	0.069 ± 0.026	-4.90	Evening
A_24_P234871	A_24_P234871	0.8098	0.119 ± 0.037	-4.87	Evening
C13orf34	A_23_P25626	0.8098	0.087 ± 0.038	-2.80	Evening
PCSK4	A_23_P16648	0.8097	0.125 ± 0.051	6.62	Morning
ARF4	A_23_P431789	0.8097	0.198 ± 0.04	-3.51	Evening
ECSIT	A_23_P119295	0.8093	0.085 ± 0.034	0.06	Evening
PDHX	A_23_P36266	0.8090	0.188 ± 0.06	-1.00	Evening

CYP4V2	A_24_P945228	0.8088	0.181 ± 0.061	-5.40	Evening
A_24_P24724	A_24_P24724	0.8087	0.169 ± 0.068	-2.21	Evening
NFAT5	A_23_P359647	0.8087	0.051 ± 0.022	1.47	Evening
CENPBD1	A_23_P206454	0.8087	0.101 ± 0.048	-0.33	Evening
A_24_P367063	A_24_P367063	0.8086	0.147 ± 0.05	4.18	Morning
NUDT8	A_23_P52569	0.8083	0.121 ± 0.046	-2.09	Evening
CLMN	A_23_P25706	0.8083	0.195 ± 0.092	-3.49	Evening
NPAS2	CPID_99/A_23_P218597	0.8082	1.054 ± 0.132	0.80	Evening
LOC51152	A_23_P149441	0.8081	0.278 ± 0.138	9.27	Morning
EIF3C	A_23_P77572	0.8081	0.104 ± 0.041	-1.25	Evening
LOC100128164	A_24_P892612	0.8079	0.322 ± 0.103	-3.89	Evening
CLUAP1	A_23_P77714	0.8079	0.084 ± 0.036	17.66	Evening
GPAM	A_23_P104237	0.8078	0.485 ± 0.115	16.22	Evening
GDE1	A_23_P54758	0.8077	0.218 ± 0.065	-1.84	Evening
ACP6	A_23_P160240	0.8076	0.274 ± 0.116	-2.17	Evening
CMAH	A_23_P351467	0.8075	0.195 ± 0.071	8.13	Morning
EXOSC6	A_32_P121794	0.8075	0.139 ± 0.044	11.11	Morning
NTN4	A_23_P204630	0.8075	0.183 ± 0.054	2.88	Morning
A_24_P15823	A_24_P15823	0.8074	0.361 ± 0.098	-2.72	Evening
SYNM	A_24_P104407	0.8073	0.214 ± 0.06	-3.05	Evening
ZNF91	A_23_P209146	0.8073	0.156 ± 0.045	6.49	Morning
FKBP7	A_23_P79622	0.8073	0.152 ± 0.065	-0.71	Evening
A_24_P912871	A_24_P912871	0.8072	0.345 ± 0.086	-1.90	Evening
AGAP3	A_23_P111452	0.8072	0.083 ± 0.027	6.53	Morning
ZNF577	A_23_P309207	0.8071	0.217 ± 0.091	3.06	Morning
PPP1R11	A_23_P19437	0.8071	0.211 ± 0.068	-1.00	Evening
BMS1	A_24_P622186	0.8071	0.153 ± 0.078	-0.47	Evening
BCL2A1	A_23_P321703	0.8070	0.098 ± 0.038	-0.52	Evening
TTC12	A_24_P73075	0.8070	0.282 ± 0.07	-1.24	Evening
APOM	A_24_P89426	0.8070	0.229 ± 0.081	-1.22	Evening
CR591764	A_32_P102252	0.8069	0.169 ± 0.046	-2.44	Evening
PXDN	A_24_P944570	0.8069	0.15 ± 0.064	-3.74	Evening
TTC12	A_23_P24535	0.8067	0.128 ± 0.063	-0.75	Evening
ENST00000444996	A_24_P383130	0.8066	0.46 ± 0.131	-0.73	Evening
CNPY2	A_23_P53288	0.8066	0.03 ± 0.014	-2.11	Evening
TRAPPC4	A_23_P127613	0.8065	0.072 ± 0.031	-2.44	Evening
SERHL2	A_23_P120953	0.8065	0.197 ± 0.036	4.93	Morning
PTPN3	A_23_P403898	0.8065	0.277 ± 0.057	6.19	Morning
ATP6V1E2	A_23_P143047	0.8064	0.153 ± 0.076	0.12	Evening
A_24_P763655	A_24_P763655	0.8064	0.251 ± 0.056	-3.17	Evening
HERC2P2	A_32_P211080	0.8064	0.251 ± 0.098	9.89	Morning
ANKRD20A2	A_24_P64241	0.8063	0.238 ± 0.1	9.54	Morning
A_24_P98145	A_24_P98145	0.8062	0.306 ± 0.093	6.68	Morning
SNTA1	A_24_P322709	0.8060	0.232 ± 0.049	-4.36	Evening
A_24_P160920	A_24_P160920	0.8059	0.29 ± 0.1	-0.25	Evening
C1orf88	A_32_P215700	0.8059	0.294 ± 0.092	2.31	Evening

ALDH4A1	A_23_P170337	0.8057	0.134 ± 0.048	17.28	Evening
ADRA1A	A_23_P8938	0.8057	0.186 ± 0.094	-2.77	Evening
RP3-377H14.5	A_32_P76811	0.8056	0.239 ± 0.072	-0.75	Evening
CERK	A_23_P211659	0.8054	0.155 ± 0.068	17.97	Evening
A_24_P324214	A_24_P324214	0.8053	0.389 ± 0.116	-0.36	Evening
ASPSCR1	A_23_P32320	0.8052	0.147 ± 0.042	-1.58	Evening
RXRA	A_23_P219176	0.8052	0.176 ± 0.043	-3.56	Evening
HDAC5	A_24_P125283	0.8052	0.108 ± 0.031	7.08	Morning
hCG_17955	A_32_P173744	0.8051	0.362 ± 0.133	-2.14	Evening
SPARC	A_23_P7642	0.8051	0.157 ± 0.059	-3.46	Evening
SLC39A11	A_23_P158969	0.8051	0.171 ± 0.069	-4.22	Evening
PPIG	A_23_P154411	0.8050	0.134 ± 0.059	8.27	Morning
KDM4C	A_24_P605233	0.8049	0.103 ± 0.049	7.49	Morning
DHCR24	A_23_P217820	0.8049	0.388 ± 0.181	-1.20	Evening
AF116713	A_24_P136905	0.8049	0.33 ± 0.14	14.53	Evening
C9orf123	A_32_P134846	0.8047	0.097 ± 0.036	-0.16	Evening
TYRO3	A_23_P54517	0.8045	0.12 ± 0.048	-3.59	Evening
P4HTM	A_23_P113317	0.8044	0.08 ± 0.026	-3.02	Evening
PPIA	A_24_P246943	0.8042	0.196 ± 0.059	-3.19	Evening
DERL1	A_23_P216043	0.8038	0.164 ± 0.053	-3.32	Evening
LOC284288	A_24_P203502	0.8038	0.348 ± 0.087	-3.54	Evening
CCDC36	A_23_P362770	0.8037	0.279 ± 0.097	12.68	Morning
CSRP2	A_23_P44724	0.8036	0.32 ± 0.055	-4.39	Evening
TMEM25	A_23_P203115	0.8035	0.163 ± 0.056	-0.40	Evening
MPND	A_23_P130886	0.8035	0.162 ± 0.045	-1.69	Evening
FIG4	A_23_P145541	0.8034	0.245 ± 0.062	-2.56	Evening
RRAGD	A_23_P133691	0.8034	0.234 ± 0.072	-2.38	Evening
PER3	CPID_448/A_24_P291231	0.8032	1.119 ± 0.12	7.41	Morning
CLEC18B	A_23_P206501	0.8031	0.168 ± 0.065	7.78	Morning
PER3	CPID_460/A_24_P291231	0.8031	1.615 ± 0.169	7.90	Morning
C6orf141	A_23_P386398	0.8031	0.152 ± 0.075	8.40	Morning
FAM171A1	A_23_P44964	0.8031	0.121 ± 0.049	-4.01	Evening
CR609342	A_32_P53107	0.8028	0.214 ± 0.062	7.60	Morning
KIF25	A_23_P59410	0.8027	0.422 ± 0.135	10.91	Morning
GNA12	A_23_P215265	0.8024	0.353 ± 0.06	-3.86	Evening
DHFR	A_23_P167553	0.8023	0.173 ± 0.07	-3.96	Evening
A_24_P578641	A_24_P578641	0.8023	0.17 ± 0.059	-2.41	Evening
A_24_P101352	A_24_P101352	0.8022	0.238 ± 0.11	0.63	Evening
AB019564	A_24_P42453	0.8021	0.248 ± 0.044	8.34	Morning
BC008001	A_23_P324011	0.8020	0.297 ± 0.122	10.12	Morning
NFE2L1	A_23_P78302	0.8019	0.136 ± 0.054	-3.84	Evening
LOC100128355	A_24_P204474	0.8017	0.277 ± 0.122	-0.89	Evening
A_24_P910372	A_24_P910372	0.8017	0.474 ± 0.134	8.30	Morning
CCHCR1	A_23_P145330	0.8017	0.131 ± 0.037	9.07	Morning
GOLGA6L6	A_24_P50972	0.8014	0.413 ± 0.095	9.18	Morning
KIAA0892	A_32_P8361	0.8014	0.22 ± 0.048	9.16	Morning

RNF150	A_24_P350589	0.8013	0.207 ± 0.068	0.41	Evening
C16orf88	A_23_P66158	0.8013	0.334 ± 0.065	3.61	Morning
PXMP2	A_23_P124122	0.8013	0.205 ± 0.072	-0.12	Evening
TACC2	A_23_P127186	0.8012	0.079 ± 0.038	6.00	Morning
GOSR2	A_23_P311608	0.8007	0.343 ± 0.091	-3.06	Evening
A_32_P118010	A_32_P118010	0.8007	0.316 ± 0.098	9.83	Morning
ABCC1	A_24_P227831	0.8007	0.119 ± 0.047	14.02	Evening
HRASLS2	A_24_P364263	0.8006	0.218 ± 0.079	-1.65	Evening
EXOC7	A_23_P100556	0.8005	0.169 ± 0.032	-3.55	Evening
DTX1	A_24_P290751	0.8004	0.56 ± 0.095	0.13	Evening
ENST00000340195	A_24_P75979	0.8003	0.261 ± 0.104	-2.93	Evening
A_32_P46456	A_32_P46456	0.8003	0.424 ± 0.138	10.34	Morning
U69195	A_24_P796652	0.8002	0.19 ± 0.083	0.24	Evening
GCAT	A_23_P40657	0.8001	0.187 ± 0.064	-1.01	Evening
FAHD2A	A_32_P81357	0.8001	0.169 ± 0.032	-1.41	Evening
ACOX3	A_23_P316381	0.8001	0.09 ± 0.039	9.77	Morning

List of circadian transcripts ordered by their fit (R^2) to a sinusoidal function. The 837 transcripts correspond to 727 unique genes, as some genes were targeted by multiple probes. The table indicates rhythm amplitude (± SE of the amplitude), timing of rhythm acrophase relative to dim light melatonin onset (DLMO), and whether the transcript was classified as peaking during morning or evening in our bioinformatic analyses.

Supplementary Table S2 – Circadian genes in both human and mouse white adipose tissue

Gene	**Acrophase in human white subcutaneous adipose tissue (Hours relative to the DLMO)**	**Acrophase in mouse white adipose tissue (Circadian Time, CT)**
EHD1	-5.3	21
OSGIN2	-5.2	2
HSPB8	-4.5	18
SNTA1	-4.4	19
TK2	-4.2	8
PLCE1	-4.1	18
STON1-GTF2A1L	-3.7	21
CLMN	-3.5	10
DYNLL2	-3.2	20
ADRA1A	-2.8	22
NQO2	-2.1	23
MME	-1.9	21
ATXN3	-1.9	14
TIMM10	-1.2	19
ARNTL	-0.9	23
DTX1	0.1	10
NPAS2	1	0
CABLES1	2.9	21
LRRC36	3.7	20
TACC2	6	10
NR1D1	6.1	8
CPSF1	6.2	8
USP6	6.5	(Usp32) 0
NR1D2	7.7	10
PER3	8.8	12
GSTT2	8.9	10
HLF	10.2	12
REV1	10.6	13
PER2	10.8	14
PER1	11.1	12
FMO2	13.8	18
DGAT2	15.6	21

Acrophase times are presented relative to dim light melatonin onset (DLMO) in humans and circadian time in mice, where CT12 is the onset of locomotor activity in continuous darkness. Where a human gene has multiple probes within the microarray, the acrophase presented is the average of all transcripts from the same gene.

Supplementary Table S3 – species overlap in GO biological processes associated with the human adipose rhythmic genes

Human_AND_Mouse_only

ID	Name	count in rhythmic mouse	frequency in biological process annotated rhythmic mouse genes	frequency in rhythmic mouse	count in rhythmic human	frequency in biological process annotated rhythmic human genes	frequency in rhythmic human
GO:0006099	tricarboxylic acid cycle	4	0.546448087	0.50441362	37	8.830548926	5.08940853
GO:0006397	mRNA processing	19	2.595628415	2.39596469	22	5.250596659	3.0261348
GO:0000184	nuclear-transcribed mRNA catabolic process, nonsense-mediated decay	4	0.546448087	0.50441362	19	4.534606205	2.61348006
GO:0008380	RNA splicing	20	2.732240437	2.5220681	18	4.295942721	2.47592847
GO:0006888	ER to Golgi vesicle-mediated transport	20	2.732240437	2.5220681	17	4.057279236	2.33837689
GO:0048012	hepatocyte growth factor receptor signaling pathway	4	0.546448087	0.50441362	17	4.057279236	2.33837689
GO:0031175	neuron projection development	17	2.322404372	2.14375788	16	3.818615752	2.20082531
GO:0006637	acyl-CoA metabolic process	6	0.819672131	0.75662043	15	3.579952267	2.06327373
GO:0042493	response to drug	27	3.68852459	3.40479193	15	3.579952267	2.06327373
GO:0006979	response to oxidative stress	14	1.912568306	1.76544767	14	3.341288783	1.92572215

Human_AND_Baboon_only

ID	Name	count in rhythmic human	frequency in biological process annotated rhythmic human genes	frequency in rhythmic human	count in rhythmic baboon	frequency in biological process annotated rhythmic baboon genes	frequency in rhythmic baboon
GO:0061025	membrane fusion	25	5.966587112	3.4387895	6	2.197802198	1.6759777
GO:0006625	protein targeting to peroxisome	12	2.863961814	1.650619	1	0.366300366	0.2793296
GO:0006406	mRNA export from nucleus	7	1.670644391	0.9628611	1	0.366300366	0.2793296
GO:0006635	fatty acid beta-oxidation	6	1.431980907	0.8253095	1	0.366300366	0.2793296
GO:0050776	regulation of immune response	6	1.431980907	0.8253095	1	0.366300366	0.2793296
GO:0031124	mRNA 3'-end processing	4	0.954653938	0.5502063	1	0.366300366	0.2793296
GO:0032204	regulation of telomere maintenance	4	0.954653938	0.5502063	1	0.366300366	0.2793296
GO:0044267	cellular protein metabolic process	4	0.954653938	0.5502063	1	0.366300366	0.2793296
GO:0048477	oogenesis	4	0.954653938	0.5502063	1	0.366300366	0.2793296
GO:1903078	positive regulation of protein localization to plasma membrane	4	0.954653938	0.5502063	1	0.366300366	0.2793296

Human_AND_Baboon_AND_Mouse

ID	Name	count in rhythmic mouse	frequency in biological process annotated rhythmic mouse genes	frequency in rhythmic mouse	count in rhythmic human	frequency in biological process annotated rhythmic human genes	frequency in rhythmic human	count in rhythmic baboon	frequency in biological process annotated rhythmic baboon genes	frequency in rhythmic baboon
GO:0055114	oxidation-reduction process	125	17.07650273	15.7629256	136	32.45823389	18.7070151	26	9.523809524	7.2625698
GO:0006355	regulation of transcription, DNA-templated	170	23.22404372	21.4375788	98	23.38902148	13.480055	92	33.6996337	25.698324
GO:0006508	proteolysis	112	15.30054645	14.1235813	83	19.80906921	11.4167813	33	12.08791209	9.2178771
GO:0016192	vesicle-mediated transport	10	1.366120219	1.26103405	60	14.31980907	8.25309491	13	4.761904762	3.6312849
GO:0006413	translational initiation	6	0.819672131	0.75662043	55	13.12649165	7.565337	3	1.098901099	0.8379888
GO:0007165	signal transduction	123	16.80327869	15.5107188	54	12.88782816	7.42778542	19	6.95970696	5.3072626
GO:0015031	protein transport	34	4.644808743	4.28751576	54	12.88782816	7.42778542	12	4.395604396	3.3519553
GO:0030154	cell differentiation	67	9.153005464	8.44892812	53	12.64916468	7.29023384	6	2.197802198	1.6759777
GO:0006468	protein phosphorylation	142	19.3989071	17.9066835	49	11.69451074	6.74002751	33	12.08791209	9.2178771
GO:0016579	protein deubiquitination	37	5.054644809	4.66582598	49	11.69451074	6.74002751	1	0.366300366	0.2793296
